# Can floral nectars reduce transmission of *Leishmania*?

**DOI:** 10.1371/journal.pntd.0010373

**Published:** 2022-05-12

**Authors:** Evan C. Palmer-Young, Ryan S. Schwarz, Yanping Chen, Jay D. Evans

**Affiliations:** 1 USDA-ARS Bee Research Laboratory, Beltsville, Maryland, United States of America; 2 Department of Biology, Fort Lewis College, Durango, Colorado, United States of America; Centro de Pesquisa Gonçalo Moniz-FIOCRUZ/BA, BRAZIL

## Abstract

**Background:**

Insect-vectored *Leishmania* are responsible for loss of more disability-adjusted life years than any parasite besides malaria. Elucidation of the environmental factors that affect parasite transmission by vectors is essential to develop sustainable methods of parasite control that do not have off-target effects on beneficial insects or environmental health. Many phytochemicals that inhibit growth of sand fly-vectored *Leishmania*—which have been exhaustively studied in the search for phytochemical-based drugs—are abundant in nectars, which provide sugar-based meals to infected sand flies.

**Principle findings:**

In a quantitative meta-analysis, we compare inhibitory phytochemical concentrations for *Leishmania* to concentrations present in floral nectar and pollen. We show that nectar concentrations of several flowering plant species exceed those that inhibit growth of *Leishmania* cell cultures, suggesting an unexplored, landscape ecology-based approach to reduce *Leishmania* transmission.

**Significance:**

If nectar compounds are as effective against parasites in the sand fly gut as predicted from experiments *in vitro*, strategic planting of antiparasitic phytochemical-rich floral resources or phytochemically enriched baits could reduce *Leishmania* loads in vectors. Such interventions could provide an environmentally friendly complement to existing means of disease control.

## Author summary

*Leishmania* parasites infect over a million people each year—including over 200,000 infections with deadly visceral leishmaniasis—resulting in a greater health burden than any human parasite besides malaria. *Leishmania* infections of humans are transmitted by blood-feeding sand flies, which also consume floral nectar. Nectar contains many chemicals that inhibit *Leishmania* growth and are candidate treatments for infection of humans. However, these same compounds could also reduce infection in nectar-consuming sand flies. By combining existing data on the chemistry of nectar and sensitivity of *Leishmania* to plant compounds, we show that some floral nectars contain sufficient chemical concentrations to inhibit growth of insect-stage *Leishmania*. Our results suggest that consumption of these nectars could reduce parasite loads in sand flies and transmission of parasites to new human hosts. In contrast to insecticide-based methods of sand fly control, incorporation of antiparasitic nectar sources into landscapes and domestic settings could benefit public health without threatening beneficial insects. These findings suggest an unexplored, landscape-based approach to reduce transmission of a major neglected tropical disease worldwide.

## Introduction

Plant secondary metabolites have a long history of use against human disease and provide the basis for both traditional medicines and many modern drugs [1], including treatments for neglected tropical diseases [2]. The sand fly-vectored *Leishmania* parasites are estimated to cause disease in >2 million humans each year, with 10% of the world’s population at risk, and have a greater health burden (as measured by loss of disability-adjusted life years) than any human parasite besides malaria [3]. These infections include an estimated >0.2M cases of visceral leishmaniasis, which, if untreated, results in >90% patient mortality [3,4]. Due to their clinical significance, *Leishmania* spp. have been studied intensively in a search for affordable and effective treatments for human infections [5], including exhaustive testing of plant extracts and their components against both mammal- and insect-associated parasite life stages [2,6]. These studies have suggested new treatments for trypanosomatid-associated infections of humans [7] and related parasites of beneficial insects [8,9]. As in humans, antimicrobial phytochemicals can enhance resistance to infection in plants themselves [10] and in other plant-consuming animals, including insects [11].

The diets of blood-feeding, disease-vectoring insects such as sand flies and mosquitoes include sugar-containing plant tissues as well as blood [12]. Sugar sources differentially affect not only vector survival, but also the development of parasitic *Plasmodium falciparum* malaria in *Anopheles* mosquitoes [13] and *Leishmania major* in sand flies (*Phlebotomus papatasi*) [14], with effects mediated by secondary metabolites [15,16]. Sand flies feed on plant sugars between acquisition and transmission of *Leishmania* to humans and other mammals [17], as demonstrated by caging flies with dye-infused branches, spectrophotometric detection of sugars or plant cell walls in the gut, and molecular analysis of field-collected flies showing the presence of plant DNA [18–20]. The importance of dietary sugars is evident from their effects on fly longevity. Flies survive less than a week under sugar source-poor desert conditions [21] and less than 2 weeks when reared on comparatively sugar-poor branches [22], but more than 7 weeks on 20% sucrose solution [22]. The abundance of sugar meal-inducible glucosidases expressed by the sand fly and by its *Leishmania* parasites provide additional evidence of mutual adaptation to an omnivorous lifestyle that exploits diverse plant sugars as food sources [23–25], and that sugar sources could be manipulated to control vectors and their parasites [16,22].

Although sand flies may acquire sugar meals from plant sap, fruit, or aphid- or cicada-derived honeydew [26], floral nectar appears to be a preferred food source, as evidenced by the attractiveness of flowering bushes and branches (relative to those soiled with honeydew) in a desert oasis [12]. The small (<1 μL) meal sizes of sand flies [23] would make the concentrated sugars found in nectar a profitable foraging resource, in spite of the small volumes available at each flower, explaining the general attractiveness of flowering plant food sources to sand flies and related dipterans [27]. The size of sugar meals is, however, impressive on a mass-specific basis—increasing the mass of females by >30% over 48 h [28]—consistent with the strong effects of meal chemistry on gut-dwelling *Leishmania*.

The role of nectar chemistry in insect disease ecology has recently been highlighted by work on infections of pollinators. Floral nectar and pollen, their constituent secondary metabolites, and the composition of flowering plant communities can ameliorate trypanosomatid growth and infection in bumble bees [8,9,29–31]. Both nectar and pollen—which may mix with and influence the chemistry of nectar at flowers [32]—contain diverse secondary metabolites that shape plant-pollinator ecology and plant-microbe ecology [33–37]. Flavonoids are one class of antimicrobial and antileishmanial compounds [38,39] that are ubiquitous in both nectar and pollen, with concentrations in pollen often exceeding 1% of total dry matter [40,41]. This suggests that consumption of secondary metabolite-rich nectars could mitigate *Leishmania* transmission by reducing infection intensity in nectar-feeding sand fly vectors [12], pointing to a new strategy for drug- and insecticide-free disease control. However, despite appreciation for the clinical antileishmanial potential of plant metabolites [2], growing recognition of the role of plant metabolites—including those in nectar and pollen—in insect infection, and the critical role of plant sugars in sand fly diets, there has been surprisingly little investigation into the potential for antileishmanial phytochemicals in the diets of sand flies to mitigate *Leishmania* transmission [14,15].

To assess the potential for floral resource-associated phytochemicals to reduce vector-borne infection, we compared phytochemical concentrations previously shown to inhibit *Leishmania* to concentrations previously found in floral nectar and pollen. Our synthesis of prior work on *Leishmania* phytochemical sensitivity with nectar and pollen secondary chemistry shows that many floral nectars contain antileishmanial compounds at concentrations sufficient to inhibit parasite growth. These findings suggest an unexplored, landscape ecology-based approach to reduce transmission of widespread and virulent *Leishmania* infections. If phytochemical concentrations that inhibit *Leishmania in vitro* are equally effective in the sand fly gut, incorporation of antiparasitic nectar sources into landscapes and domestic settings could simultaneously benefit pollinator and public health.

## Methods

We compared the flavonoid concentrations found in a previous survey of methanolic extracts from 26 floral nectars and 28 pollens [40,42] with previously published results from *in vitro* screening of various *Leishmania* spp. (Table A in S1 Text). We focused on flavonoids because these compounds were the most consistently present class of compounds across both nectar and pollen [40] and—particularly in the case of quercetin—some of the most potent and selective compounds against *Leishmania* [39,43,44]. To prevent overestimation of inhibitory potential that could result from including flavonoids of lesser or unknown antiparasitic activity, we further distinguished between total flavonoid concentrations and those with a kaempferol, quercetin, apigenin, or luteolin aglycone, each of which has well-documented antileishmanial effects [39,45,46] (Table A in S1 Text).

We analyzed micromolar concentrations to enable pooling across compounds with different parent flavonoids and glycosides. Flavonoid glycosides—including those of quercetin and kaempferol—can be less potent against *Leishmania* than are their parent aglycones [39], which can more easily cross cell membranes [47]. However, we included flavonoid glycosides because these compounds are hydrolyzed by intestinal glucosidases—a variety of which are found in sand flies [28]—to their corresponding aglycones [48,49]. These glucosidases have been shown to form antileishmanial aglycones from glycosylated coumarins in intestinal extracts [16]. We focus our discussion on nectar because sand flies, like other Diptera, do not have chewing mouthparts that would enable direct consumption of pollen and other solid foods [20]. However, incidental presence of pollen in nectar can dramatically increase the nectar’s concentrations of amino acids [50], with ecologically relevant effects on nectar-feeding insects [32]. Pollen could similarly affect nectar’s phytochemical profile and antimicrobial effects. For example, presumably pollen-derived cinnamic acid-spermidine conjugates were found in nectar of two species in our previous survey—*Digitalis purpurea* and *Helianthus annuus* [40]. In *H*. *annuus*, nectar concentrations averaged 1.7% of pollen concentrations, despite exclusion of large insects that contribute to such "contamination" [50] for 24 h prior to sampling. We therefore also discuss pollen concentrations that exceed the *Leishmania* IC50 estimates by >100-fold, on the grounds that the much (235-fold [40]) higher flavonoid concentrations found in pollen could meaningfully alter the antiparasitic activity of nectar, even when pollen accounts for <1% of nectar volume.

## Results

We compiled 18 *Leishmania* IC50 estimates for 4 flavonoids—quercetin (n = 8), kaempferol (n = 4), apigenin, and luteolin (n = 3 each) that have been relatively well studied for effects on *Leishmania* spp. cell cultures (Table A in S1 Text). Most (11 of 18) of the assays used the promastigote (i.e., insect-associated) life stage; the remainder used either intracellular (n = 4) or axenic (n = 3) amastigotes (Table A in S1 Text). These *Leishmania* IC50 estimates were then compared to the flavonoid concentrations found in a previous survey of secondary metabolites of nectar and pollen [40].

**Fig 1:**
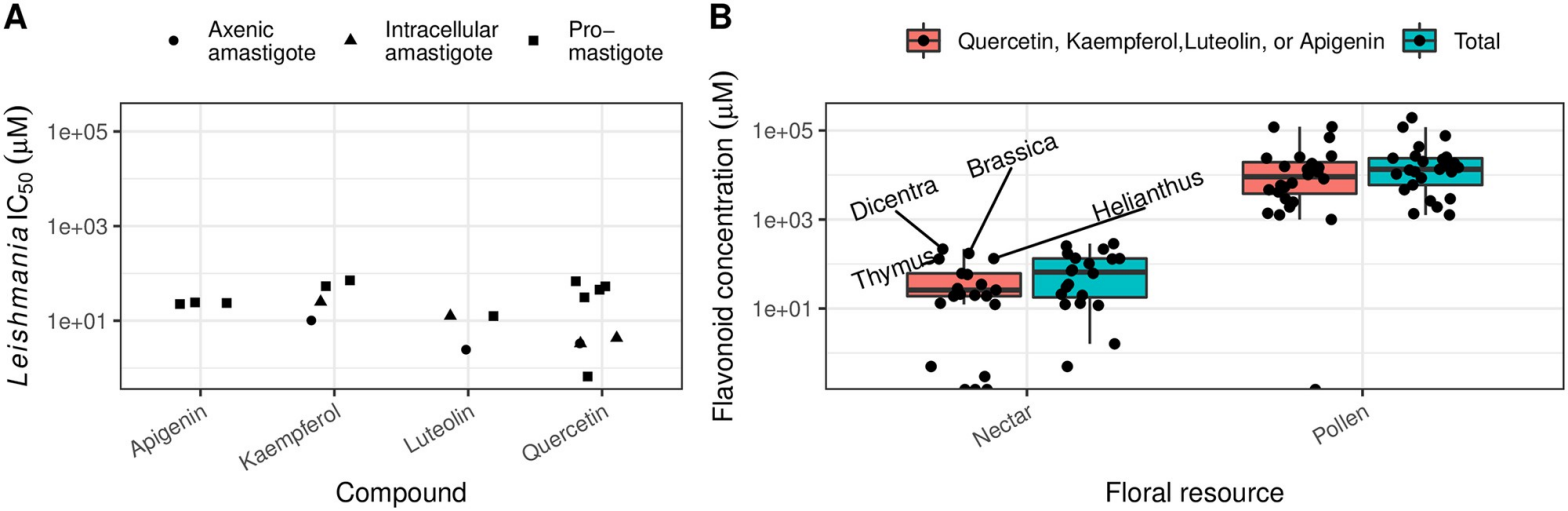
Published *Leishmania* IC50 estimates for selected flavonoids (A) relative to concentrations of the corresponding compounds in nectar and pollen (B). Shapes in panel (A) correspond to the *Leishmania* stage tested. Boxplots in panel (B) show medians and interquartile ranges for concentrations of quercetin, kaempferol, apigenin and luteolin derivatives (red boxes) and total flavonoids (blue boxes). Points show median concentrations (pooled across individual samples) by species. Text annotations denote species with >100 μM of the selected flavonoids in nectar (*Brassica napus*, *Dicentra eximia*, *Helianthus annuus*, and *Thymus vulgaris*). Literature references for *Leishmania* IC50 estimates are given in Table A in S1 Text.

Flavonoids were found in the nectar of 21 of 26 species (81%) and in pollen of 26 of 28 species (93%), accounting for 30% of the total phytochemical content in nectar and 41% in pollen [40]. Total flavonoid concentrations exceeded 100 μM in 8 of 26 nectars (31%, median concentration 30.9 μM, IQR 4.24–127 μM; median 61.4 μM after exclusion of the five species without nectar flavonoids) and exceeded 10^4^ μM in 18 of 28 pollens (64%, median 1.36 ∙ 10^4^ μM, IQR 4.31 ∙ 10^3^ to 2.30 ∙ 10^4^ μM) (Fig 1). Glycosides of quercetin (found in 9 of 26 nectars and 14 of 28 pollens) and kaempferol (5 of 26 nectars and 19 of 28 pollens) were most common [40].

Compounds with a parent aglycone of quercetin, kaempferol, apigenin, or luteolin accounted for 62% of flavonoid compounds and 54% of molar concentrations in nectar, and 72% of compounds and 75% of molar concentrations in pollen. In nectar, median concentration of this subset of compounds across all species (20.3 μM, IQR 9.37–58.9 μM) was remarkably close to the 23.1 μM median IC50 for *Leishmania* (based on 18 references (Table A in S1 Text)). Concentrations exceeded 100 μM (i.e., more than the highest *Leishmania* IC50 for any of the parent compounds) in nectar from 4 of 26 species (*Dicentra eximia*, *Brassica napus*, *Helianthus annuus*, *and Thymus vulgaris*). In pollen, median concentrations exceeded 10^4^ μM (i.e., >100-fold the greatest *Leishmania* IC50) in pollen from 12 of 28 species, including two species (*Lythrum salicaria* (1.21 ∙ 10^5^) and *Solidago canadensis* (1.19 ∙ 10^5^)) with concentrations >10^5^ μM—over three orders of magnitude above the greatest *Leishmania* IC50 (Fig 1).

**Fig 2:**
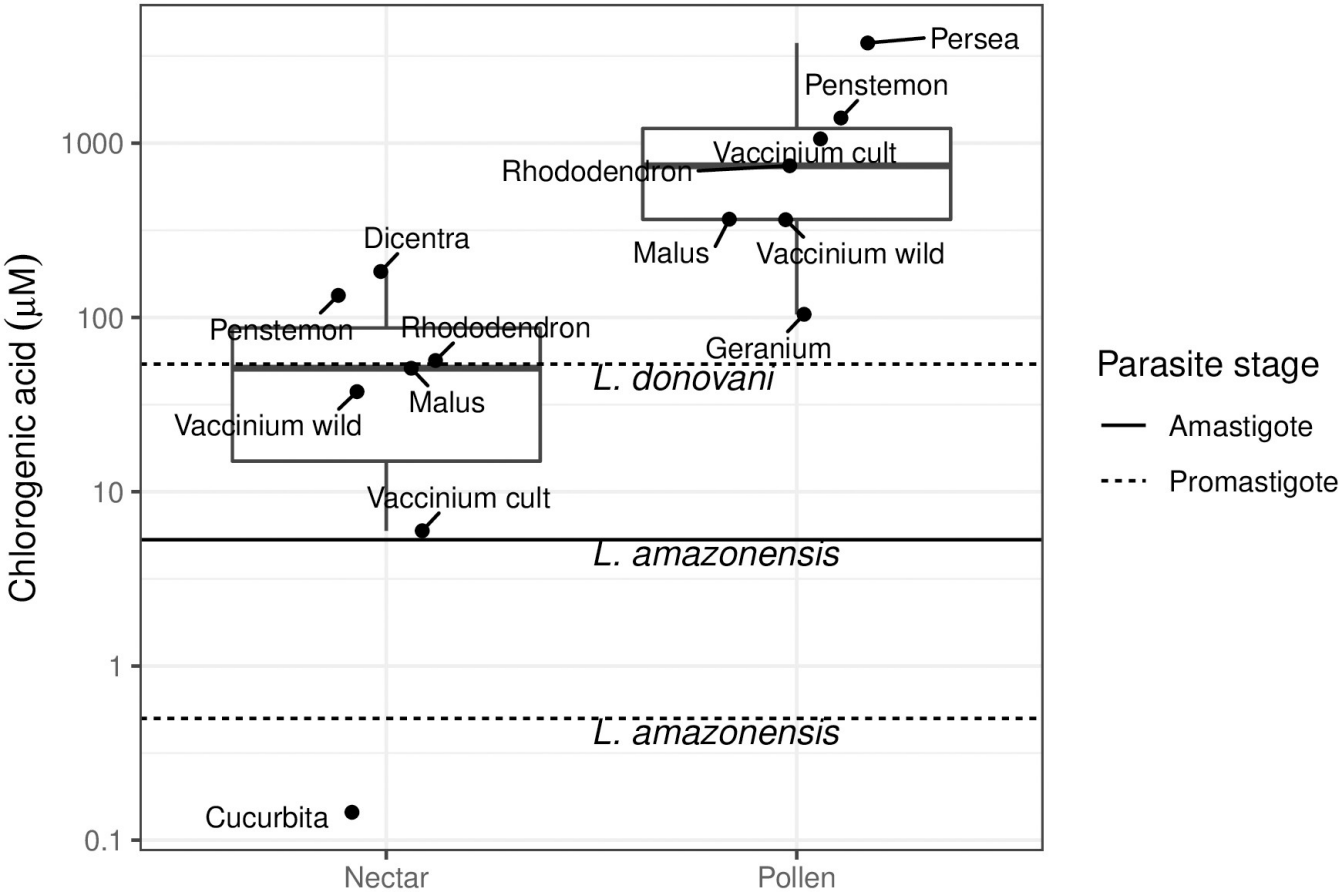
Concentrations of chlorogenic acid in nectar and pollen in comparison with inhibitory concentrations for *Leishmania*. Points represent median concentrations from species with detectable chlorogenic acid (sampled in [40]). Horizontal lines show published IC50 values [51,52]. Sampled plant species (labeled by genus) were *Dicentra eximia*, *Penstemon digitalis*, *Rhododendron prinophyllum*, *Malus domestica*, *Vaccinium corymbosum*, *Cucurbita pepo*, *Persea americana*, *and Geranium maculatum*. For *Vaccinium*, "cult" refers to cultivars and "wild" refers to wild plants.

Antileishmanial compounds in nectar were not limited to flavonoids. Seven nectars contained chlorogenic acid, with a median concentration (51.2 μM) similar to the IC50 for *L*. *donovani* promastigotes (54 μM [51]) and 100-fold greater than the IC50 for *L*. *amazonensis* promastigotes (0.5 μM [52]). The species with the highest median concentration of chlorogenic acid (*Dicentra eximia*, 184 μM) also had the highest concentration of the selected flavonoids (Fig 1). Nectar concentrations of two additional species (*Penstemon digitalis*, 134 μM) and *Rhododendron prinophyllum* (56.7 μM) also exceeded the *L*. *donovani* promastigote IC50 (Fig 2). Chlorogenic acid was also found in seven pollens at up to 3760 μM (*Persea americana*), with a median concentration (1227 μM) over 20-fold greater than that found in nectar and over three orders of magnitude above the *L*. *amazonensis* promastigote IC50 [52] (Fig 2).

**Fig 3:**
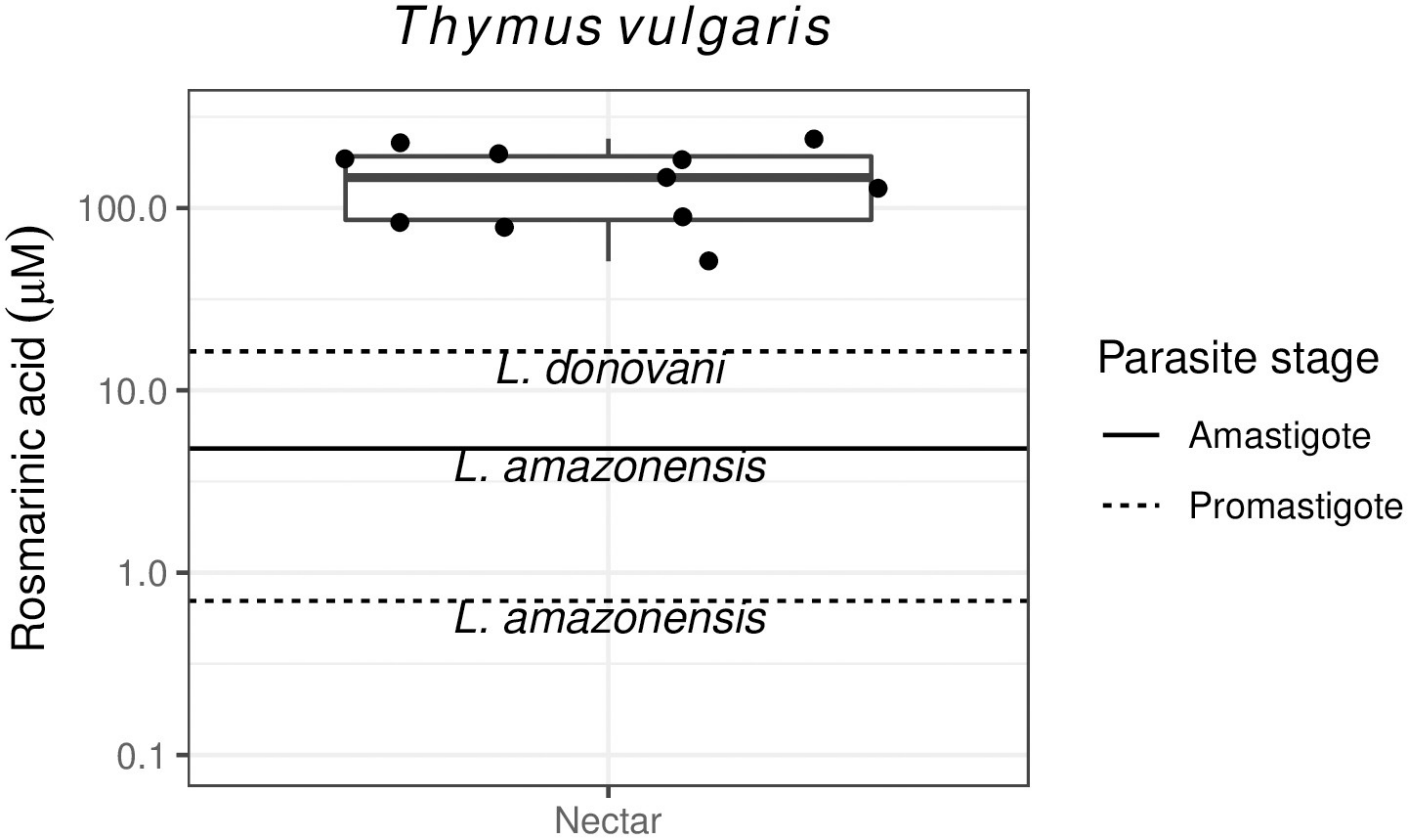
Concentrations of rosmarinic acid in *Thymus vulgaris* nectar in comparison with inhibitory concentrations for *Leishmania*. Points represent individual nectar samples (from [40]). Horizontal lines show published IC50 values [51,52].

Nectar of one species (*Thymus vulgaris*) contained the caffeic acid-dihydroxyphenyl lactic acid ester rosmarinic acid. Median concentration (165 μM, IQR 87.7–206 μM) was 10-fold greater than the IC50 for *L*. *donovani* promastigotes (16.3 μM [51])—against which rosmarinic acid and apigenin were the most selective of the compounds evaluated—over 30-fold greater than the 4.8 μM IC50 for *L*. *amazonensis* amastigotes [52], and over 200-fold greater than the 0.7 μM reported for *L*. *amazonensis* promastigotes [52] (Fig 3). Nectar of *T*. *vulgaris* is also notable for its high thymol content (26.1 μg mL^-1^ [53]), which exceeds six of the eight IC50 values reported for *Leishmania* promastigotes (Table A in S1 Text, [54,55]).

## Discussion

Our synthesis of a previous survey on the quantitative phytochemical composition of nectar and pollen with the extensive body of research on phytochemical-mediated inhibition of Leishmania *in vitro* reveals the potential for floral resources to ameliorate vector-mediated transmission of *Leishmania*. The most common compounds in nectar and pollen—flavonoids and their glycosides—have shown strong inhibitory effects against *Leishmania* [39,44,56]. Our findings indicate that a subset of the floral nectars analyzed to date—including the common garden herb *Thymus vulgaris* (thyme) and the widespread crop species *Helianthus annuus* (cultivated sunflower) contain bioactive flavonoids at concentrations that inhibit growth of diverse *Leishmania in vitro*. Incidentally, both plant species have also been shown to mitigate transmission and infectivity of bumble bee trypanosomatids, including in field mesocosms and landscape surveys [30,31]. Further investigation of the effects of specific nectar and other sugar sources on sand fly infection is needed. However, deliberate encouragement of these and other plants with trypanosomatid-inhibiting chemistry could reduce infection in disease-vectoring, hematophagous insects as well.

Our data suggest that around 20% of nectars contain flavonoids at strongly antiparasitic concentrations, although this number likely varies by region and season. Our analysis was focused on bee-pollinated species in the Northeast United States, where sand flies are absent, and therefore contained few of the specific plant species naturally used by sand flies in *Leishmania*-endemic regions. However, sand flies have been observed to prefer cultivated gardens (to which such plants could be introduced) over endemic vegetation [20], and have been associated with plants in the same taxonomic families as those represented by the species analyzed here. For example, *Brassica napus* (Brassicaceae) nectar was among the highest in flavonoids; flowers of another member of this family (*Sinapis alba*) elicited feeding by *Phlebotomus papatasi* [57]. Nectar of *Impatiens capensis* (Balsaminaceae) had flavonoid concentrations (20.9 μM, all from strongly antileishmanial compounds) close to the median of the nectars examined (20.3 μM) and the median *Leishmania* IC50 (23.1 μM); branches of the congener *I*. *balsamina* were fed upon by *Lutzomyia youngi* in Colombian coffee plantations [58]. On the other hand, nectar flavonoid concentrations were considerably lower (1.6 μM total) in *Trifolium pratense*, the only Fabaceae species tested; plants of this family have been strongly associated with sand flies in field sampling [59] and DNA metabarcoding studies [60,61]. Flavonoid concentrations were also low (<1 μM) in *Cucurbita pepo* (Cucurbitaceae) and undetectable in *Catalpa speciosa* (Bignoniaceae), two other plant families associated with sand flies [20]. Based on these results, the amounts of antileishmanial flavonoids ingested by flies could vary substantially in different landscapes.

Besides floral nectar, other known sugar sources may also possess flavonoid-mediated antileishmanial activity. Flavonoid concentrations of sand fly-attracting fruits [26] appear similar to those found in nectar. Combined quercetin and luteolin contents ranged from unquantifiable in honeydew melon to 22.8 μM in nectarine, 33.1 and 39.7 μM in red and white guava, and 53.6, 84.1, and 91.1 μM in white, black, and red grapes respectively [62]. Honeydew from sapsucking insects such as aphids [12] likely also contains types and quantities of flavonoids similar to those found in floral nectar, based on the similar flavonoid profiles of honey from these two sources [63]. Further experiments are needed to assess the chemistry of local, fly-attracting, sugar-providing plant species and their effects on insect host and parasite mortality, as demonstrated for lectin-rich plant sugar sources in Israel [14,15,22]. Given that sand fly feeding on branches [19], flowers [12], and fruits [26] tends to be highly selective on a few local species, the scope of such research is likely achievable.

We predict that our analysis—which accounts only for direct effects of a few compounds on parasites as estimated from *in vitro* studies—provides a conservative estimate of the effects of plant compounds on disease transmission. First, we focused on a limited subset of nectar components whose effects on *Leishmania* have been thoroughly studied, ignoring the effects of co-occurring chemicals that could also affect parasites (e.g., other flavonoids, lectins, and alkaloids), whether present in the ingested sugar source or formed during sand fly digestion (e.g., deglycosylation of coumarins or cyanogenic glycosides to compounds that reduce parasitic infection [16,64]). Second, these direct effects could be amplified by host-mediated reductions in levels of parasites due to phytochemical ingestion. For example, nectar-derived flavonoids stimulated immune gene expression in honey bees [65]; similar flavonoid-induced immune stimulation could enhance parasite clearance in flies. In addition, besides their effects on protozoa specifically, flavonoids are generally antimicrobial [38], and could inhibit growth of midgut bacteria that facilitate *Leishmania* infection [66]. It would be of interest to contrast the effects of similar flavonoid concentrations taken directly from plant tissues—which are delivered to the sand fly midgut—versus those from surface sugars (e.g., nectar and honeydew), which are first stored in the crop [57]. The gradual release of nectar and honeydew from the crop to into the anterior midgut [57] could limit the exposure of midgut-dwelling parasites to phytochemicals from these sources.

The effect of sugar-containing meals from plant sources was a long-overlooked component of sand fly ecology that proved crucial in *Leishmania* transmission [17]. However, feeding of sand flies on several plant taxa causes marked parasite mortality—up to 88% in the case of castor bean (*Ricinus communis*) [14], the lectins of which agglutinate a variety of insect trypanosomatids [67]—paralleling the strong effects of plant sugar sources on malaria infection in mosquitoes [13]. Although flowering plant nectar sources might at first glance appear to be a liability for *Leishmania* transmission due to the food they provide to sand flies, sugar starvation in fact results in greater vector infection intensity and natural selection for flies with lesser parasite resistance [68,69]. This finding is consistent with the preponderance of *Leishmania* hotspots in arid regions, where plant sugar sources are scarce [68,69]. High parasite loads also alter sand fly feeding on mammals in ways that promote transmission to new hosts [70]. These lines of evidence suggest that despite their role as vector food sources, phytochemical-rich floral nectar sources could have a net transmission-reducing effect.

Feeding of sand flies on floral nectar may also result in incidental pollen exposure that, due to pollen’s high flavonoid concentrations, has strong effects on *Leishmania* in the fly gut. Such incidental exposure was suggested by the high prevalence of Pinaceae DNA associated with sand flies at sites apparently lacking such plants [20]. This association was postulated to reflect exposure of flies to windblown pollen, which could also account for at least some of the DNA from *Cannabis sativa—*another wind-pollinated species not visibly present [20]. Introduction of pollen to nectaries by bees can increase nectar amino acid concentrations by an order of magnitude, and potentially introduce antiparasitic compounds from con- and heterospecific pollens as well [50]. Given that flavonoid concentrations in pollen are 200-fold higher than those in nectar [40], incidental ingestion of even small amounts of pollen could substantively inhibit proliferation of parasites and the transmission potential of their vectors. In *H*. *annuus*, pollen-associated spermidines occurred at concentrations >1% of those in pollen even when pollinators were excluded [40]. In our meta-analysis, eight of the 28 pollens previously surveyed contained flavonoids at concentrations that exceeded 100-fold the maximum inhibitory concentration reported for *Leishmania* (Fig 2). This suggests that as little as 1% incidental addition of pollen to nectaries might be sufficient for *Leishmania* inhibition, even for nectars that lack antileishmanial flavonoids initially.

Whether antiparasitic compounds are present in secreted nectars or due to incidental introduction of pollen, nectars rich in phytochemicals are promising candidates for ecological mitigation of *Leishmania* transmission. Parasites of this genus appear both sensitive to flavonoids and, given the parasite’s establishment in the midgut and forward migration in the alimentary canal [17], directly exposed to ingested compounds before appreciable metabolism of these compounds—by hosts or microbiota in the abdominal midgut—can occur. The limited intestinal absorption of ingested flavonoids [49], hydrolysis of glycosides found in plants to their more potent aglycones in the intestine [28,49], and likelihood of direct contact between parasites in the anterior midgut and ingested phytochemicals all indicate the potential for flavonoid-rich nectars to reduce *Leishmania* infection in sand flies. However, empirical testing of these compounds in sand fly diets is necessary to confirm their efficacy in the insect vector and model the effects of sugar sources on parasite infection, vector longevity, and disease transmission, as was recently done for malaria [13]. In addition, the broader ecological effects of floral compounds—whose effects are unlikely to be limited to sand flies alone—must be considered before implementation of interventions, particularly those that involve introduction of non-endemic plant species.

## Conclusions

The global toll of *Leishmania* infection and the difficulties of eradicating its sand fly vectors and non-human reservoirs demand the development of new, environmentally compatible strategies to reduce parasite transmission [71]. Our synthesis of existing data shows that sugar-seeking sand flies are attracted to floral resources, and that floral nectars contain antileishmanial phytochemicals at concentrations that inhibit replication of parasite cell cultures. The extent to which floral resources influence *Leishmania* epidemiology will depend on the contribution of nectar to sand fly diets and the extent to which *in vitro* inhibitory effects are realized in the guts of infected flies. If the effects of nectar on insect infection are commensurate with predictions based on nectar phytochemistry, reduction of transmission *via* supply of antiparasitic nectar sources in local landscapes—or phytochemical-based, transmission-blocking baits [16]–could positively influence public health. Such interventions could reduce reliance on drug treatments that may be costly, inaccessible, or potentially hazardous [3] while simultaneously supporting populations of beneficial insects and their resistance to insect-specific trypanosomatid infections [29,30]. The fields of insect ecology and medicinal chemistry for insect-vectored parasites have thus far developed more in parallel than in concert. Integrating knowledge of medicinal plant chemistry and plant-mediated tritrophic interactions that affect parasites in disease-vectoring insects holds promise for environmentally friendly control of trypanosomatid threats to global health.

## Supporting information

S1 Text Supplementary Table A, references, and metadata. (PDF)

S1 Data Zipped folder with data spreadsheets for *Leishmania* inhibitory concentrations (leishmania_ic50) and nectar and pollen flavonoid concentrations (nectar.pollen.flavonoids). (ZIP)

## References

[1] Schmeller T, Wink M. Utilization of Alkaloids in Modern Medicine. In: Roberts MF, Wink M, editors. Alkaloids: Biochemistry, Ecology, and Medicinal Applications. Boston, MA: Springer US; 1998. pp. 435–459. doi: 10.1007/978-1-4757-2905-4_18

[2] Le TB, Beaufay C, Bonneau N, Mingeot-Leclercq M-P, Quetin-Leclercq J. Anti-protozoal activity of essential oils and their constituents against *Leishmania*, *Plasmodium* and Trypanosoma. Phytochimie. 2018;18: 1–33. http://hdl.handle.net/2078.1/199166

[3] McGwire BS, Satoskar AR. Leishmaniasis: clinical syndromes and treatment. QJM Int J Med. 2014;107: 7–14. doi: 10.1093/qjmed/hct116 23744570 PMC3869292

[4] Steverding D. The history of leishmaniasis. Parasit Vectors. 2017;10: 82. doi: 10.1186/s13071-017-2028-5 28202044 PMC5312593

[5] De Rycker M, Hallyburton I, Thomas J, Campbell L, Wyllie S, Joshi D, et al. Comparison of a High-Throughput High-Content Intracellular *Leishmania donovani* Assay with an Axenic Amastigote Assay. Antimicrob Agents Chemother. 2013;57: 2913–2922. doi: 10.1128/AAC.02398-12 23571538 PMC3697379

[6] Anthony J-P, Fyfe L, Smith H. Plant active components–a resource for antiparasitic agents? Trends Parasitol. 2005;21: 462–468. doi: 10.1016/j.pt.2005.08.004 16099722

[7] Brindha J, Balamurali MM, Chanda K. An Overview on the Therapeutics of Neglected Infectious Diseases—Leishmaniasis and Chagas Diseases. Front Chem. 2021;9: 622286. doi: 10.3389/fchem.2021.622286 33777895 PMC7994601

[8] Richardson LL, Adler LS, Leonard AS, Andicoechea J, Regan KH, Anthony WE, et al. Secondary metabolites in floral nectar reduce parasite infections in bumblebees. Proc R Soc Lond B Biol Sci. 2015;282: 20142471. doi: 10.1098/rspb.2014.2471 25694627 PMC4345440

[9] Palmer-Young EC, Sadd BM, Stevenson PC, Irwin RE, Adler LS. Bumble bee parasite strains vary in resistance to phytochemicals. Sci Rep. 2016;6: 37087. doi: 10.1038/srep37087 27883009 PMC5121629

[10] Hartmann T. From waste products to ecochemicals: Fifty years research of plant secondary metabolism. Phytochemistry. 2007;68: 2831–2846. doi: 10.1016/j.phytochem.2007.09.017 17980895

[11] de Roode JC, Lefèvre T, Hunter MD, Lefevre T, Hunter MD. Self-medication in animals. Science. 2013;340: 150–151. doi: 10.1126/science.1235824 23580516

[12] Müller G, Schlein Y. Nectar and honeydew feeding of *Phlebotomus papatasi* in a focus of *Leishmania major* in Neot Hakikar oasis. J Vector Ecol. 2004;29: 154–158. 15266752

[13] Hien DF d S, Dabiré KR, Roche B, Diabaté A, Yerbanga RS, Cohuet A, et al. Plant-Mediated Effects on Mosquito Capacity to Transmit Human Malaria. PLOS Pathog. 2016;12: e1005773. doi: 10.1371/journal.ppat.1005773 27490374 PMC4973987

[14] Schlein Y, Jacobson RL. Mortality of *Leishmania major* in *Phlebotomus papatasi* Caused by Plant Feeding of the Sand Flies. Am J Trop Med Hyg. 1994;50: 20–27. doi: 10.4269/ajtmh.1994.50.1.TM0500010020 8304569

[15] Jacobson RL, Schlein Y. Lectins and toxins in the plant diet of *Phlebotomus papatasi* (Diptera: Psychodidae) can kill *Leishmania major* promastigotes in the sandfly and in culture. Ann Trop Med Parasitol. 1999;93: 351–356. doi: 10.1080/00034989958348 10656036

[16] Ferreira TN, Pita-Pereira D, Costa SG, Brazil RP, Moraes CS, Díaz-Albiter HM, et al. Transmission blocking sugar baits for the control of *Leishmania* development inside sand flies using environmentally friendly beta-glycosides and their aglycones. Parasit Vectors. 2018;11: 614. doi: 10.1186/s13071-018-3122-z 30501613 PMC6271627

[17] Adler S, Theodor O. Transmission of Disease Agents by Phlebotomine Sand Flies. Annu Rev Entomol. 1957;2: 203–226. doi: 10.1146/annurev.en.02.010157.001223

[18] Schlein Y, Muller G. Assessment of Plant Tissue Feeding by Sand Flies (Diptera: Psychodidae) and Mosquitoes (Diptera: Culicidae). J Med Entomol. 1995;32: 882–887. doi: 10.1093/jmedent/32.6.882 8551514

[19] Junnila A, Müller GC, Schlein Y. Identification of plant tissues from the gut of *Phlebotomus papatasi* by DNA analysis. Acta Trop. 2011;117: 14–18. doi: 10.1016/j.actatropica.2010.08.019 20849806

[20] Abbasi I, de Queiroz ATL, Kirstein OD, Nasereddin A, Horwitz BZ, Hailu A, et al. Plant-feeding phlebotomine sand flies, vectors of leishmaniasis, prefer *Cannabis sativa*. Proc Natl Acad Sci. 2018;115: 11790–11795. doi: 10.1073/pnas.1810435115 30373823 PMC6243281

[21] Schlein Y, Jacobson RL. Sugar meals and longevity of the sandfly *Phlebotomus papatasi* in an arid focus of *Leishmania major* in the Jordan Valley. Med Vet Entomol. 1999;13: 65–71. doi: 10.1046/j.1365-2915.1999.00138.x 10194751

[22] Schlein Y, Jacobson RL, Müller GC. Sand fly feeding on noxious plants: a potential method for the control of leishmaniasis. Am J Trop Med Hyg. 2001;65: 300–303. doi: 10.4269/ajtmh.2001.65.300 11693873

[23] Dillon RJ, El Kordy E. Carbohydrate Digestion in Sandflies: α-Glucosidase Activity in the Midgut of *Phlebotomus langeroni*. Comp Biochem Physiol B Biochem Mol Biol. 1997;116: 35–40. doi: 10.1016/s0305-0491(96)00195-2 9162544

[24] Gontijo NF, Melo MN, Riani EB, Almeida-Silva S, Mares-Guia ML. Glycosidases in *Leishmania* and Their Importance for *Leishmania* in Phlebotomine Sandflies with Special Reference to Purification and Characterization of a Sucrase. Exp Parasitol. 1996;83: 117–124. doi: 10.1006/expr.1996.0055 8654540

[25] Jacobson R, Schlein Y, Eisenberger C. The biological function of sand fly and *Leishmania* glycosidases. Med Microbiol Immunol (Berl). 2001;190: 51–55. doi: 10.1007/s004300100079 11770110

[26] Junnila A, Müller GC, Schlein Y. Attraction of *Phlebotomus papatasi* to common fruit in the field. J Vector Ecol. 2011;36: S206–S211. doi: 10.1111/j.1948-7134.2011.00132.x 21366776

[27] Müller GC, Revay EE, Schlein Y. Relative attraction of the sand fly Phlebotomus papatasi to local flowering plants in the Dead Sea region. J Vector Ecol. 2011;36: S187–S194. doi: 10.1111/j.1948-7134.2011.00130.x 21366774

[28] Jacobson RL, Studentsky L, Schlein Y. Glycolytic and chitinolytic activities of *Phlebotomus papatasi* (Diptera: Psychodidae) from diverse ecological habitats. Folia Parasitol (Praha). 2013;54: 301–309. doi: 10.14411/fp.2007.039 18303772

[29] Koch H, Woodward J, Langat MK, Brown MJF, Stevenson PC. Flagellum Removal by a Nectar Metabolite Inhibits Infectivity of a Bumblebee Parasite. Curr Biol. 2019;29: 3494–3500.e5. doi: 10.1016/j.cub.2019.08.037 31607528

[30] Adler LS, Barber NA, Biller OM, Irwin RE. Flowering plant composition shapes pathogen infection intensity and reproduction in bumble bee colonies. Proc Natl Acad Sci. 2020;117: 11559–11565. doi: 10.1073/pnas.2000074117 32393622 PMC7261119

[31] Giacomini JJ, Leslie J, Tarpy DR, Palmer-Young EC, Irwin RE, Adler LS. Medicinal value of sunflower pollen against bee pathogens. Sci Rep. 2018;8: 14394. doi: 10.1038/s41598-018-32681-y 30258066 PMC6158195

[32] Erhardt A, Baker I. Pollen amino acids—an additional diet for a nectar feeding butterfly? Plant Syst Evol. 1990;169: 111–121. doi: 10.1007/BF00935989

[33] Junker RR, Keller A. Microhabitat heterogeneity across leaves and flower organs promotes bacterial diversity. FEMS Microbiol Ecol. 2015;91: fiv097. doi: 10.1093/femsec/fiv097 26253507

[34] Rivest S, Forrest JRK. Defence compounds in pollen: why do they occur and how do they affect the ecology and evolution of bees? New Phytol. 2020;225: 1053–1064. doi: 10.1111/nph.16230 31569278

[35] Huang M, Sanchez-Moreiras AM, Abel C, Sohrabi R, Lee S, Gershenzon J, et al. The major volatile organic compound emitted from *Arabidopsis thaliana* flowers, the sesquiterpene (*E*)-β-caryophyllene, is a defense against a bacterial pathogen. New Phytol. 2012;193: 997–1008. doi: 10.1111/j.1469-8137.2011.04001.x 22187939

[36] Heil M. Nectar: generation, regulation and ecological functions. Trends Plant Sci. 2011;16: 191–200. doi: 10.1016/j.tplants.2011.01.003 21345715

[37] Stevenson PC. For antagonists and mutualists: the paradox of insect toxic secondary metabolites in nectar and pollen. Phytochem Rev. 2020;19: 603–614. doi: 10.1007/s11101-019-09642-y

[38] Cushnie TPT, Lamb AJ. Antimicrobial activity of flavonoids. Int J Antimicrob Agents. 2005;26: 343–356. doi: 10.1016/j.ijantimicag.2005.09.002 16323269 PMC7127073

[39] Tasdemir D, Kaiser M, Brun R, Yardley V, Schmidt TJ, Tosun F, et al. Antitrypanosomal and antileishmanial activities of flavonoids and their analogues: *in vitro*, *in vivo*, structure-activity relationship, and quantitative structure-activity relationship studies. Antimicrob Agents Chemother. 2006;50: 1352–64. doi: 10.1128/AAC.50.4.1352-1364.2006 16569852 PMC1426963

[40] Palmer-Young EC, Farrell IW, Adler LS, Milano NJ, Egan PA, Junker RR, et al. Chemistry of floral rewards: intra- and interspecific variability of nectar and pollen secondary metabolites across taxa. Ecol Monogr. 2019;89: e01335. doi: 10.1002/ecm.1335

[41] Serra Bonvehi J, Soliva Torrentó M, Centelles Lorente E. Evaluation of polyphenolic and flavonoid compounds in honeybee-collected pollen produced in Spain. J Agric Food Chem. 2001;49: 1848–1853. doi: 10.1021/jf0012300 11308335

[42] Palmer-Young EC, Farrell IW, Adler LS, Milano NJ, Egan PA, Irwin RE, et al. Secondary metabolites from nectar and pollen: a resource for ecological and evolutionary studies. Ecology. 2019;100: e02621. doi: 10.1002/ecy.2621 30667044

[43] Fonseca-Silva F, Inacio JDF, Canto-Cavalheiro MM, Almeida-Amaral EE. Reactive Oxygen Species Production and Mitochondrial Dysfunction Contribute to Quercetin Induced Death in *Leishmania amazonensis*. PLOS ONE. 2011;6: e14666. doi: 10.1371/journal.pone.0014666 21346801 PMC3035610

[44] Fonseca-Silva F, Inacio JDF, Canto-Cavalheiro MM, Almeida-Amaral EE. Reactive Oxygen Species Production by Quercetin Causes the Death of *Leishmania amazonensis* Intracellular Amastigotes. J Nat Prod. 2013;76: 1505–1508. doi: 10.1021/np400193m 23876028

[45] Mittra B, Saha A, Roy Chowdhury A, Pal C, Mandal S, Mukhopadhyay S, et al. Luteolin, an Abundant Dietary Component is a Potent Anti-leishmanial Agent that Acts by Inducing Topoisomerase II-mediated Kinetoplast DNA Cleavage Leading to Apoptosis. Mol Med. 2000;6: 527–541. doi: 10.1007/BF03401792 10972088 PMC1949962

[46] Fonseca-Silva F, Canto-Cavalheiro MM, Menna-Barreto RFS, Almeida-Amaral EE. Effect of Apigenin on *Leishmania amazonensis* Is Associated with Reactive Oxygen Species Production Followed by Mitochondrial Dysfunction. J Nat Prod. 2015;78: 880–884. doi: 10.1021/acs.jnatprod.5b00011 25768915

[47] Xiao J. Dietary flavonoid aglycones and their glycosides: Which show better biological significance? Crit Rev Food Sci Nutr. 2017;57: 1874–1905. doi: 10.1080/10408398.2015.1032400 26176651

[48] Walle T, Otake Y, Walle UK, Wilson FA. Quercetin glucosides are completely hydrolyzed in ileostomy patients before absorption. J Nutr. 2000;130: 2658–2661. doi: 10.1093/jn/130.11.2658 11053503

[49] Hostetler GL, Ralston RA, Schwartz SJ. Flavones: Food Sources, Bioavailability, Metabolism, and Bioactivity. Adv Nutr. 2017;8: 423–435. doi: 10.3945/an.116.012948 28507008 PMC5421117

[50] Willmer PG. The effects of insect visitors on nectar constituents in temperate plants. Oecologia. 1980;47: 270–277. doi: 10.1007/BF00346832 28309483

[51] Antwi CA, Amisigo CM, Adjimani JP, Gwira TM. In vitro activity and mode of action of phenolic compounds on *Leishmania donovani*. PLoS Negl Trop Dis. 2019;13: e0007206. doi: 10.1371/journal.pntd.0007206 30802252 PMC6405172

[52] Montrieux E, Perera WH, García M, Maes L, Cos P, Monzote L. In vitro and in vivo activity of major constituents from *Pluchea carolinensis* against *Leishmania amazonensis*. Parasitol Res. 2014;113: 2925–2932. doi: 10.1007/s00436-014-3954-1 24906989

[53] Rothchild KW, Adler LS, Irwin RE, Sadd BM, Stevenson PC, Palmer-Young EC. Effects of short-term exposure to naturally occurring thymol concentrations on transmission of a bumble bee parasite. Ecol Entomol. 2018;43: 567–577. doi: 10.1111/een.12631

[54] Silva ARST, Scher R, Santos FV, Ferreira SR, Cavalcanti SCH, Correa CB, et al. Leishmanicidal Activity and Structure-Activity Relationships of Essential Oil Constituents. Molecules. 2017;22: 815. doi: 10.3390/molecules22050815 28509873 PMC6154737

[55] de Morais SM, Vila-Nova NS, Bevilaqua CML, Rondon FC, Lobo CH, de Alencar Araripe Noronha Moura A, et al. Thymol and eugenol derivatives as potential antileishmanial agents. Bioorg Med Chem. 2014;22: 6250–6255. doi: 10.1016/j.bmc.2014.08.020 25281268 PMC5106290

[56] Muzitano MF, Cruz EA, de Almeida AP, Da Silva SAG, Kaiser CR, Guette C, et al. Quercitrin: an antileishmanial flavonoid glycoside from *Kalanchoe pinnata*. Planta Med. 2006;72: 81–83. doi: 10.1055/s-2005-873183 16450304

[57] Schlein Y, Warburg A. Phytophagy and the Feeding Cycle of *Phlebotomus Papatasi* (Diptera: Psychodidae) Under Experimental Conditions1. J Med Entomol. 1986;23: 11–15. doi: 10.1093/jmedent/23.1.11 3950922

[58] Alexander B, Usma MC. Potential sources of sugar for the phlebotomine sandfly *Lutzomyia youngi* (Diptera: Psychodidae) in a Colombian coffee plantation. Ann Trop Med Parasitol. 1994;88: 543–549. doi: 10.1080/00034983.1994.11812902 7979645

[59] Cameron MM, Pessoa F a. C, Vasconcelos AW, Ward RD. Sugar meal sources for the phlebotomine sandfly *Lutzomyia longipalpis* in Ceará State, Brazil. Med Vet Entomol. 1995;9: 263–272. doi: 10.1111/j.1365-2915.1995.tb00132.x 7548943

[60] de Lima LHGM, Mesquita MR, Skrip L, de Souza Freitas MT, Silva VC, Kirstein OD, et al. DNA barcode for the identification of the sand fly *Lutzomyia longipalpis* plant feeding preferences in a tropical urban environment. Sci Rep. 2016;6: 29742. doi: 10.1038/srep29742 27435430 PMC4951712

[61] Hassaballa IB, Sole CL, Cheseto X, Torto B, Tchouassi DP. Afrotropical sand fly-host plant relationships in a leishmaniasis endemic area, Kenya. PLoS Negl Trop Dis. 2021;15: e0009041. doi: 10.1371/journal.pntd.0009041 33556068 PMC7895382

[62] Haytowitz DB, Wu X, Bhagwat S. USDA Database for the Flavonoid Content of Selected Foods, Release 3.3. US Dep Agric Beltsville MD USA. 2018.

[63] Nešović M, Gašić U, Tosti T, Trifković J, Baošić R, Blagojević S, et al. Physicochemical analysis and phenolic profile of polyfloral and honeydew honey from Montenegro. RSC Adv. 2020;10: 2462–2471. doi: 10.1039/C9RA08783D 35496084 PMC9048719

[64] Ferreira ME, Rojas de Arias A, Yaluff G, de Bilbao NV, Nakayama H, Torres S, et al. Antileishmanial activity of furoquinolines and coumarins from *Helietta apiculata*. Phytomedicine. 2010;17: 375–378. doi: 10.1016/j.phymed.2009.09.009 19879121

[65] Mao W, Schuler MA, Berenbaum MR. Honey constituents up-regulate detoxification and immunity genes in the western honey bee *Apis mellifera*. Proc Natl Acad Sci U S A. 2013;110: 8842–8846. doi: 10.1073/pnas.1303884110 23630255 PMC3670375

[66] Louradour I, Monteiro CC, Inbar E, Ghosh K, Merkhofer R, Lawyer P, et al. The midgut microbiota plays an essential role in sand fly vector competence for *Leishmania major*. Cell Microbiol. 2017;19: e12755. doi: 10.1111/cmi.12755 28580630 PMC5587349

[67] Petry K, Schottelius J, Dollet M. Differentiation of *Phytomonas* sp. and lower trypanosomatids (*Herpetomonas*, *Crithidia*) by agglutination tests with lectins. Parasitol Res. 1987;74: 1–4. doi: 10.1007/BF00534923

[68] Schlein Y, Jacobson RL. Hunger tolerance and *Leishmania* in sandflies. Nature. 2001;414: 168–168. doi: 10.1038/35102679 11700547

[69] Schlein Y, Jacobson RL. Linkage between susceptibility of *Phlebotomus papatasi* to *Leishmania major* and hunger tolerance. Parasitology. 2002;125: 343–348. doi: 10.1017/s0031182002002147 12403322

[70] Molyneux DH, Jefferies D. Feeding behaviour of pathogen-infected vectors. Parasitology. 1986;92: 721–736. doi: 10.1017/s0031182000065574 3737249

[71] Garlapati R, Iniguez E, Serafim TD, Mishra PK, Rooj B, Sinha B, et al. Towards a Sustainable Vector-Control Strategy in the Post Kala-Azar Elimination Era. Front Cell Infect Microbiol. 2021;11: 114. doi: 10.3389/fcimb.2021.641632 33768013 PMC7985538

