# Supplementary material for: Can floral nectars reduce transmission of Leishmania?
Source: PLoS Negl Trop Dis. 2022 May 12;16(5):e0010373. doi: 10.1371/journal.pntd.0010373 (PMC9098005; doi:10.1371/journal.pntd.0010373)
Supplement: S1 Text — (PDF) [file pntd.0010373.s001.pdf]

Supplementary information:

Can floral nectars reduce transmission of *Leishmania?*

Running title: Nectar phytochemicals and *Leishmania* transmission

Evan C Palmer-Young [1]*, Ryan S Schwarz [2], Jay D Evans [1]

[1] USDA-ARS Bee Research Lab, Beltsville, MD, USA

[2] Department of Biology, Fort Lewis College, Durango, CO, USA

*Corresponding author: ecp52@cornell.edu

Contents

Supplementary tables 3

Supplementary data 7

References 8

SUPPLEMENTARY TABLES

Supplementary Table A. Reported inhibitory concentrations (IC50) for the flavonoids apigenin, luteolin, kaempferol, and quercetin; and the esters chlorogenic and rosmarinic acids. Species are listed as in the original publications, but note that *L. chagasi* is considered synonymous with *L. infantum* [1]. Inhibitory concentrations for amastigotes (primarily found in mammals) are included for completeness. However, note that the promastigote stage is the predominant form in the gut of sand flies.

Substance	Species	Stage	IC50 (µg/mL)	Assay	Duration (h)	Reference	Notes
Apigenin	*Leishmania amazonensis*	P	6.4	Cell counts	24	[2]	
Apigenin	*Leishmania mexicana*	P	6.6	Resazurin fluorescence	72	[3]	
Apigenin	*Leishmania donovani*	P	6.1	MTT absorbance	72	[4]	
Chlorogenic acid	*Leishmania amazonensis*	P	0.2	MTT absorbance	72	[5]	
Chlorogenic acid	*Leishmania amazonensis*	IA	1.9	Cell counts	48	[5]	
Chlorogenic acid	*Leishmania donovani*	P	19.1	MTT absorbance	72	[4]	
Kaempferol	*Leishmania donovani*	AA	2.9	Resazurin fluorescence	72	[6]	
Kaempferol	*Leishmania donovani*	IA	7.15	Cell counts	72	[7]	
Kaempferol	*Leishmania peruviana*	P	20.4	Cell counts	72	[8]	
Kaempferol	*Leishmania braziliensis*	P	15.3	Cell counts	72	[8]	
Luteolin	*Leishmania donovani*	P	3.6	Cell counts	24	[9]	
Luteolin	*Leishmania donovani*	IA	3.6	Cell counts	24	[9]	
Luteolin	*Leishmania donovani*	AA	0.7	Resazurin fluorescence	72	[6]	
Quercetin	*Leishmania amazonensis*	P	9.4	Cell counts	48	[10]	
Quercetin	*Leishmania amazonensis*	P	0.2	MTT absorbance	72	[5]	

Quercetin	*Leishmania amazonensis*	IA	1.3	Cell counts	48	[5]	
Quercetin	*Leishmania amazonensis*	IA	1	Cell counts	72	[11]	
Quercetin	*Leishmania donovani*	P	13.7	Cell counts	24	[9]	
Quercetin	*Leishmania donovani*	AA	1	Resazurin fluorescence	72	[6]	
Quercetin	*Leishmania peruviana*	P	16.1	Cell counts	72	[8]	
Quercetin	*Leishmania braziliensis*	P	20.7	Cell counts	72	[8]	
Rosmarinic acid	*Leishmania amazonensis*	P	0.2	MTT absorbance	72	[5]	
Rosmarinic acid	*Leishmania amazonensis*	IA	1.7	Cell counts	48	[5]	
Rosmarinic acid	*Leishmania donovani*	P	5.9	MTT absorbance	72	[4]	
Thymol	*Leishmania amazonensis*	P	26.8	Resazurin fluorescence	24	[12]	
Thymol	*Leishmania infantum*	P	12.85	Bio-luminescence	24	[13]	
Thymol	*Leishmania infantum*	IA	23.93	ELISA	48	[13]	
Thymol	*Leishmania chagasi*	P	9.8	MTT absorbance	72	[14]	
Thymol	*Leishmania chagasi*	P	9.8	MTT absorbance	72	[15]	
Thymol	*Leishmania infantum*	P	7.2	MTT absorbance	24	[16]	
Thymol	*Leishmania amazonensis*	P	19.5	Cell counts	48	[17]	
Thymol	*Leishmania chagasi*	P	65.2	Cell counts	72	[18]	

Abbreviations:

P: Promastigote

AA: Axenic amastigote

IA: Intracellular amastigote

MTS: [3-(4, 5 dimethyl-thiazol-2-yl) 5- (3-carboxymethoxyphenyl)-2-(4-sulphonyl)-2H-tetrazolium]"

PMS: phenazine methosulfate

MTT: 3-[4,5-dimethylthiazol-2-yl]-2,5-diphenyltetrazolium bromide

SUPPLEMENTARY DATA

Zipped folder with data spreadsheets for *Leishmania* inhibitory concentrations (**leishmania_ic50**) and nectar and pollen flavonoid concentrations (**nectar.pollen.flavonoids**).

REFERENCES

1. Steverding D. The history of leishmaniasis. Parasit Vectors. 2017;10: 82. doi:10.1186/s13071-017-2028-5

2. Fonseca-Silva F, Canto-Cavalheiro MM, Menna-Barreto RFS, Almeida-Amaral EE. Effect of Apigenin on *Leishmania amazonensis* Is Associated with Reactive Oxygen Species Production Followed by Mitochondrial Dysfunction. J Nat Prod. 2015;78: 880–884. doi:10.1021/acs.jnatprod.5b00011

3. Alotaibi A, Ebiloma GU, Williams R, Alfayez IA, Natto MJ, Alenezi S, et al. Activity of Compounds from Temperate Propolis against *Trypanosoma brucei* and *Leishmania mexicana*. Molecules. 2021;26: 3912. doi:10.3390/molecules26133912

4. Antwi CA, Amisigo CM, Adjimani JP, Gwira TM. In vitro activity and mode of action of phenolic compounds on *Leishmania donovani*. PLoS Negl Trop Dis. 2019;13: e0007206. doi:10.1371/journal.pntd.0007206

5. Montrieux E, Perera WH, García M, Maes L, Cos P, Monzote L. In vitro and in vivo activity of major constituents from *Pluchea carolinensis* against *Leishmania amazonensis*. Parasitol Res. 2014;113: 2925–2932. doi:10.1007/s00436-014-3954-1

6. Tasdemir D, Kaiser M, Brun R, Yardley V, Schmidt TJ, Tosun F, et al. Antitrypanosomal and antileishmanial activities of flavonoids and their analogues: *in vitro*, *in vivo*, structure-activity relationship, and quantitative structure-activity relationship studies. Antimicrob Agents Chemother. 2006;50: 1352–64. doi:10.1128/AAC.50.4.1352-1364.2006

7. Halder A, Das S, Bera T, Mukherjee A. Rapid synthesis for monodispersed gold nanoparticles in kaempferol and anti-leishmanial efficacy against wild and drug resistant strains. RSC Adv. 2017;7: 14159–14167. doi:10.1039/C6RA28632A

8. Marín C, Boutaleb-Charki S, Díaz JG, Huertas O, Rosales MJ, Pérez-Cordon G, et al. Antileishmaniasis Activity of Flavonoids from *Consolida oliveriana*. J Nat Prod. 2009;72: 1069–1074. doi:10.1021/np8008122

9. Mittra B, Saha A, Roy Chowdhury A, Pal C, Mandal S, Mukhopadhyay S, et al. Luteolin, an Abundant Dietary Component is a Potent Anti-leishmanial Agent that Acts by Inducing Topoisomerase II-mediated Kinetoplast DNA Cleavage Leading to Apoptosis. Mol Med. 2000;6: 527–541. doi:10.1007/BF03401792

10. Fonseca-Silva F, Inacio JDF, Canto-Cavalheiro MM, Almeida-Amaral EE. Reactive Oxygen Species Production and Mitochondrial Dysfunction Contribute to Quercetin Induced Death in *Leishmania amazonensis*. PLOS ONE. 2011;6: e14666. doi:10.1371/journal.pone.0014666

11. Fonseca-Silva F, Inacio JDF, Canto-Cavalheiro MM, Almeida-Amaral EE. Reactive Oxygen Species Production by Quercetin Causes the Death of *Leishmania amazonensis* Intracellular Amastigotes. J Nat Prod. 2013;76: 1505–1508. doi:10.1021/np400193m

12. Silva ARST, Scher R, Santos FV, Ferreira SR, Cavalcanti SCH, Correa CB, et al. Leishmanicidal Activity and Structure-Activity Relationships of Essential Oil Constituents. Molecules. 2017;22: 815. doi:10.3390/molecules22050815

13. de Morais SM, Vila-Nova NS, Bevilaqua CML, Rondon FC, Lobo CH, de Alencar Araripe Noronha Moura A, et al. Thymol and eugenol derivatives as potential antileishmanial agents. Bioorg Med Chem. 2014;22: 6250–6255. doi:10.1016/j.bmc.2014.08.020

14. de Melo JO, Bitencourt TA, Fachin AL, Cruz EMO, de Jesus HCR, Alves PB, et al. Antidermatophytic and antileishmanial activities of essential oils from *Lippia gracilis* Schauer genotypes. Acta Trop. 2013;128: 110–115. doi:10.1016/j.actatropica.2013.06.024

15. Farias-Junior PA, Rios MC, Moura TA, Almeida RP, Alves PB, Blank AF, et al. Leishmanicidal activity of carvacrol-rich essential oil from *Lippia sidoides* Cham. Biol Res. 2012;45: 399–402. doi:10.4067/S0716-97602012000400012

16. Youssefi MR, Moghaddas E, Tabari MA, Moghadamnia AA, Hosseini SM, Farash BRH, et al. In Vitro and In Vivo Effectiveness of Carvacrol, Thymol and Linalool against *Leishmania infantum*. Molecules. 2019;24: 2072. doi:10.3390/molecules24112072

17. de Medeiros M das GF, da Silva AC, Citó AM das GL, Borges AR, de Lima SG, Lopes JAD, et al. In vitro antileishmanial activity and cytotoxicity of essential oil from *Lippia sidoides* Cham. Parasitol Int. 2011;60: 237–241. doi:10.1016/j.parint.2011.03.004

18. Escobar P, Milena Leal S, Herrera LV, Martinez JR, Stashenko E. Chemical composition and antiprotozoal activities of Colombian *Lippia* spp essential oils and their major components. Mem Inst Oswaldo Cruz. 2010;105: 184–190. doi:10.1590/S0074-02762010000200013
