# Supplementary material for: Can floral nectars reduce transmission of Leishmania?
Source: PLoS Negl Trop Dis. 2022 May 12;16(5):e0010373. doi: 10.1371/journal.pntd.0010373 (PMC9098005; doi:10.1371/journal.pntd.0010373)
Supplement: S1 Data — (ZIP) [file pntd.0010373.s002.zip › S1_data_references_v0.docx]

# References

1. Steverding D. The history of leishmaniasis. Parasit Vectors. 2017;10: 82. doi:10.1186/s13071-017-2028-5

2. Fonseca-Silva F, Canto-Cavalheiro MM, Menna-Barreto RFS, Almeida-Amaral EE. Effect of Apigenin on *Leishmania amazonensis* Is Associated with Reactive Oxygen Species Production Followed by Mitochondrial Dysfunction. J Nat Prod. 2015;78: 880–884. doi:10.1021/acs.jnatprod.5b00011

3. Alotaibi A, Ebiloma GU, Williams R, Alfayez IA, Natto MJ, Alenezi S, et al. Activity of Compounds from Temperate Propolis against *Trypanosoma brucei* and *Leishmania mexicana*. Molecules. 2021;26: 3912. doi:10.3390/molecules26133912

4. Antwi CA, Amisigo CM, Adjimani JP, Gwira TM. In vitro activity and mode of action of phenolic compounds on *Leishmania donovani*. PLoS Negl Trop Dis. 2019;13: e0007206. doi:10.1371/journal.pntd.0007206

5. Montrieux E, Perera WH, García M, Maes L, Cos P, Monzote L. In vitro and in vivo activity of major constituents from *Pluchea carolinensis* against *Leishmania amazonensis*. Parasitol Res. 2014;113: 2925–2932. doi:10.1007/s00436-014-3954-1

6. Tasdemir D, Kaiser M, Brun R, Yardley V, Schmidt TJ, Tosun F, et al. Antitrypanosomal and antileishmanial activities of flavonoids and their analogues: *in vitro*, *in vivo*, structure-activity relationship, and quantitative structure-activity relationship studies. Antimicrob Agents Chemother. 2006;50: 1352–64. doi:10.1128/AAC.50.4.1352-1364.2006

7. Halder A, Das S, Bera T, Mukherjee A. Rapid synthesis for monodispersed gold nanoparticles in kaempferol and anti-leishmanial efficacy against wild and drug resistant strains. RSC Adv. 2017;7: 14159–14167. doi:10.1039/C6RA28632A

8. Marín C, Boutaleb-Charki S, Díaz JG, Huertas O, Rosales MJ, Pérez-Cordon G, et al. Antileishmaniasis Activity of Flavonoids from *Consolida oliveriana*. J Nat Prod. 2009;72: 1069–1074. doi:10.1021/np8008122

9. Mittra B, Saha A, Roy Chowdhury A, Pal C, Mandal S, Mukhopadhyay S, et al. Luteolin, an Abundant Dietary Component is a Potent Anti-leishmanial Agent that Acts by Inducing Topoisomerase II-mediated Kinetoplast DNA Cleavage Leading to Apoptosis. Mol Med. 2000;6: 527–541. doi:10.1007/BF03401792

10. Fonseca-Silva F, Inacio JDF, Canto-Cavalheiro MM, Almeida-Amaral EE. Reactive Oxygen Species Production and Mitochondrial Dysfunction Contribute to Quercetin Induced Death in *Leishmania amazonensis*. PLOS ONE. 2011;6: e14666. doi:10.1371/journal.pone.0014666

11. Fonseca-Silva F, Inacio JDF, Canto-Cavalheiro MM, Almeida-Amaral EE. Reactive Oxygen Species Production by Quercetin Causes the Death of *Leishmania amazonensis* Intracellular Amastigotes. J Nat Prod. 2013;76: 1505–1508. doi:10.1021/np400193m

12. Silva ARST, Scher R, Santos FV, Ferreira SR, Cavalcanti SCH, Correa CB, et al. Leishmanicidal Activity and Structure-Activity Relationships of Essential Oil Constituents. Molecules. 2017;22: 815. doi:10.3390/molecules22050815

13. de Morais SM, Vila-Nova NS, Bevilaqua CML, Rondon FC, Lobo CH, de Alencar Araripe Noronha Moura A, et al. Thymol and eugenol derivatives as potential antileishmanial agents. Bioorg Med Chem. 2014;22: 6250–6255. doi:10.1016/j.bmc.2014.08.020

14. de Melo JO, Bitencourt TA, Fachin AL, Cruz EMO, de Jesus HCR, Alves PB, et al. Antidermatophytic and antileishmanial activities of essential oils from *Lippia gracilis* Schauer genotypes. Acta Trop. 2013;128: 110–115. doi:10.1016/j.actatropica.2013.06.024

15. Farias-Junior PA, Rios MC, Moura TA, Almeida RP, Alves PB, Blank AF, et al. Leishmanicidal activity of carvacrol-rich essential oil from *Lippia sidoides* Cham. Biol Res. 2012;45: 399–402. doi:10.4067/S0716-97602012000400012

16. Youssefi MR, Moghaddas E, Tabari MA, Moghadamnia AA, Hosseini SM, Farash BRH, et al. In Vitro and In Vivo Effectiveness of Carvacrol, Thymol and Linalool against *Leishmania infantum*. Molecules. 2019;24: 2072. doi:10.3390/molecules24112072

17. de Medeiros M das GF, da Silva AC, Citó AM das GL, Borges AR, de Lima SG, Lopes JAD, et al. In vitro antileishmanial activity and cytotoxicity of essential oil from *Lippia sidoides* Cham. Parasitol Int. 2011;60: 237–241. doi:10.1016/j.parint.2011.03.004

18. Escobar P, Milena Leal S, Herrera LV, Martinez JR, Stashenko E. Chemical composition and antiprotozoal activities of Colombian *Lippia* spp essential oils and their major components. Mem Inst Oswaldo Cruz. 2010;105: 184–190. doi:10.1590/S0074-02762010000200013
