# Supplementary material for: Can floral nectars reduce transmission of Leishmania?
Source: PLoS Negl Trop Dis. 2022 May 12;16(5):e0010373. doi: 10.1371/journal.pntd.0010373 (PMC9098005; doi:10.1371/journal.pntd.0010373)
Supplement: Attachment — Submitted filename: leishmania_hypothesis_responses_v1.1.pdf [file pntd.0010373.s003.pdf]

Responses to reviewer comments

Many thanks to each of the three reviewers for their constructive comments, which have helped us to improve the manuscript. The original comments are listed in italics, followed by our responses and, when applicable, quotation of the text changed in the manuscript and its location. Line numbers refer to the unmarked version of the manuscript, rather than the one with changes tracked.

Methods

Reviewer #1: As this is a meta-analysis there is no testable hypothesis. So the other questions are irrelevant

Reviewer #2: This section methods is fine for me except some points to address. Please find below my comments
1- Line 101-102 (Ref 27), could you explain how flavonoid glycosides are less effective than aglycones?

Response: Flavonoid aglycones generally have greater potency than do glycosides due to the aglycones' smaller size and comparatively hydrophobic structure. We have now better explained the empirical and theoretical basis for this statement in the text: "Flavonoid glycosides—including those of quercetin and kaempferol—can be less potent against *Leishmania* than are their parent aglycones [27], which can more easily cross cell membranes [35]." **(Line 104)**

Reviewer #3: The study design was clear, however it is not strong enough support the hypothesis raised in this study.

Response: We have added several qualifying statements to emphasize the preliminary nature of our results and the need to test the effectiveness of phytochemicals in parasitized sand flies:

In the last section of the Abstract: "**Significance:** If nectar compounds are as effective against parasites in the sand fly gut as predicted from experiments *in vitro*, strategic planting of antiparasitic phytochemical-rich floral resources or phytochemically enriched baits could reduce *Leishmania* loads in vectors." **(Line 25)**

At the end of the Introduction: "If phytochemical concentrations that inhibit *Leishmania in vitro* are equally effective in the sand fly gut, incorporation of antiparasitic nectar sources into landscapes and domestic settings could simultaneously benefit pollinator and public health." **(Line 89)**

In the beginning of the Discussion: "Further investigation of the effects of specific nectar and other sugar sources on sand fly infection is needed…" **(Line 175)**

In conclusion to the Discussion: "However, empirical testing of these compounds in sand fly diets is necessary to confirm their efficacy in the insect vector and model the effects of sugar sources on parasite infection, vector longevity, and disease transmission." **(Line 259)**

Results

Reviewer #1: As this is a compilation of other scientists' research there was no analysis plan to begin with. The results are clearly stated.

Figures:
Figure 1 A: There are no values even close to 1,000 (1e+03), so why have a graph that goes to 1e+05 (100,000)?

Response: It is correct that none of the values in 1A exceed 10^3. However, we scaled the y-axis to match that of the graph of nectar and pollen concentrations in Figure 1B.

1B: Is very busy and hard to read – can it be simplified?

Response: We have removed the mapping of point shape to flavonoid category in panel B. This simplifies panel B and avoids confusion with the shape key in panel A, in which shape corresponds to parasite life stage.

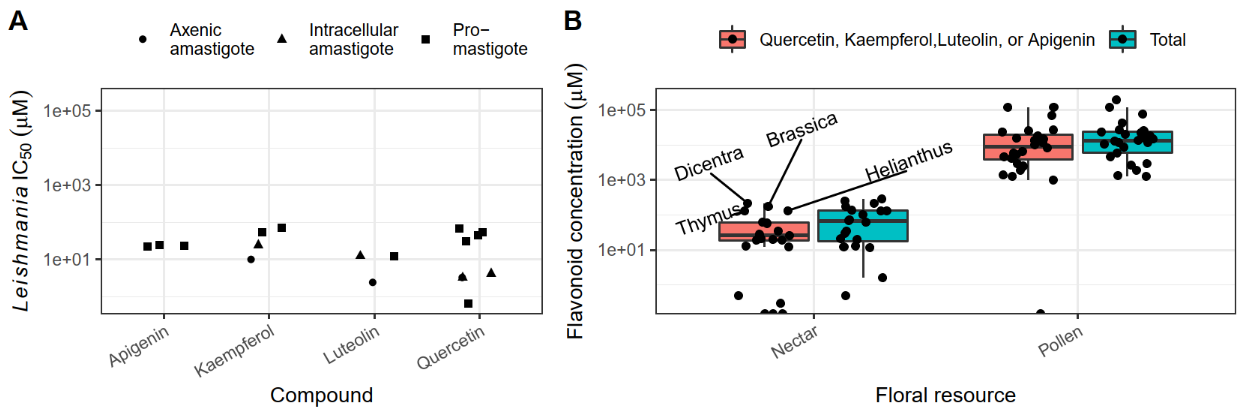

Figures 2 & 3. N.B. fig. 2 uses 1e + scale, while fig 3 uses 0.1 to 100. The second is so much easier to read.

Response: Thanks for the constructive advice. We have now changed the y-axis scaling of Figure 2 to match the (preferred) format of Figure 3.

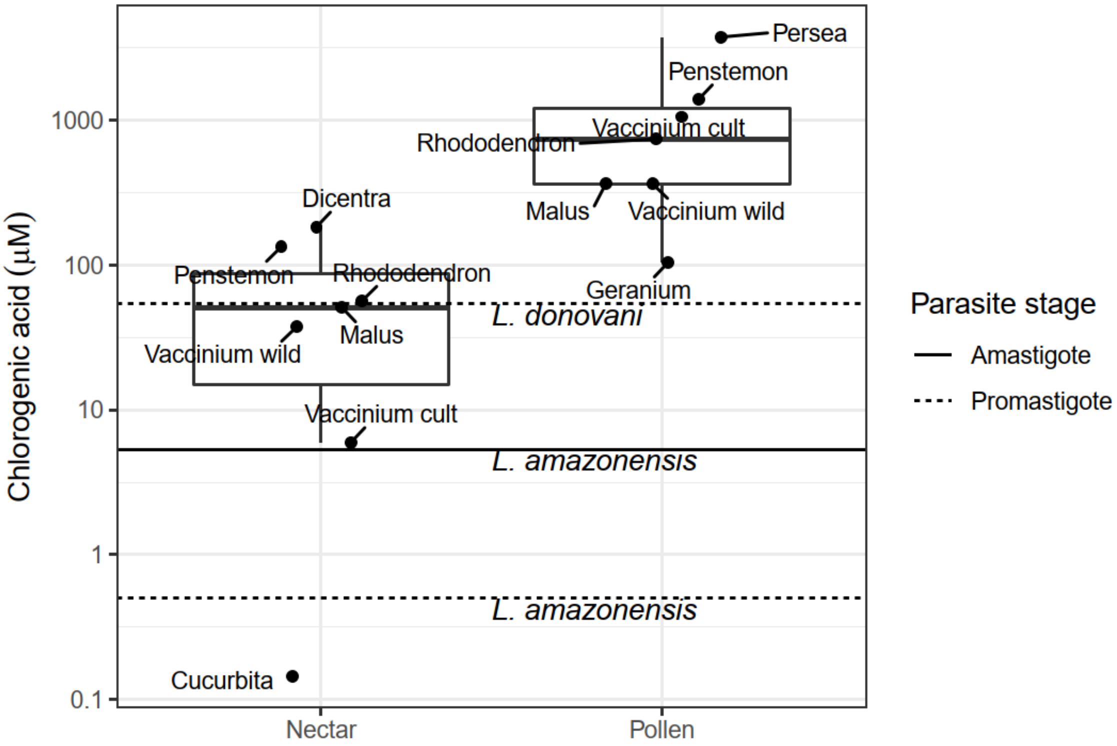

Table
Table includes data on amastigotes, a stage in the life cycle of the parasite that is very transitory in the sand fly.

Response: This is a fair point. We have clarified the legend for Supplementary Table S1 to state that: "Inhibitory concentrations for amastigotes (primarily found in mammals) are included for completeness. However, note that the promastigote stage is the predominant form in the gut of sand flies."

Reviewer #2: Good, but is the concentration of floral resources that you use the same concentration that could Phlebotomus ingest in the wild?

Response: We focused our analysis on concentrations found in floral nectar, which is one of the preferred sugar sources for *Phlebotomus* (Muller and Schlein 2004, J. Vector Ecology) and was the resource for which we had the most knowledge of secondary chemistry, based on our recent characterization article. However, in the Discussion, we have now added a new paragraph on the potential flavonoid-mediated activity of other sugar sources as well:

"Besides floral nectar, other known sugar sources may also possess flavonoid-mediated antileishmanial activity. Flavonoid concentrations of sand fly-attracting fruits [16] appear similar to those found in nectar. Combined quercetin and luteolin contents ranged from unquantifiable in honeydew melon to 22.8 µM in nectarine, 33.1 39.7 µM in red and white guava, and 53.6, 84.1, and 91.1 µM in white, black, and red grapes respectively [53]. Honeydew from sapsucking insects such as aphids [12] likely also contains types and quantities of flavonoids similar to those found in floral nectar, based on the similar flavonoid profiles of honey from these two sources [54]." **(beginning Line 197)**

Reviewer #3: Concentrations evaluated here are based only in vitro exposition of the parasite to compounds, ignoring their effect during Leishmania-sand fly interactions, i.e., ignoring the behavior and physiology of the insect host, in which is essential to the potential for the vector control.

Response: This is an important point. We have devoted a new section to potential plant-fly-parasite interactions, including modulation of immune function and microbiota, in the expanded Discussion:

"We predict that our analysis—which accounts only for direct effects of a few compounds on parasites as estimated from *in vitro* studies—provides a conservative estimate of the effects of plant compounds on disease transmission. First, we focused on a limited subset of nectar components whose effects on *Leishmania* have been thoroughly studied, ignoring the effects of co-occurring chemicals that could also affect parasites. Second, these direct effects could be amplified by host-mediated reductions in levels of parasites due to phytochemical ingestion. For example, nectar-derived flavonoids stimulated immune gene expression in honey bees [57]; similar flavonoid-induced immune stimulation could enhance parasite clearance in flies. In addition, besides their effects on protozoa specifically, flavonoids are generally antimicrobial [26], and could inhibit growth of midgut bacteria that facilitate *Leishmania* infection [58]. It would be of interest to contrast the effects of similar flavonoid concentrations taken directly from plant tissues—which are delivered to the midgut—versus those from surface sugars (e.g., nectar and honeydew), which are first stored in the crop [48]. These differences in digestion could affect the phytochemical exposure of parasites found in different gut regions." **(beginning Line 208)**

Conclusions

Reviewer #1: The conclusions are adequate, but the limitations are (i.e. lack of field data) not mentioned.
No evidence is presented that sand flies actually feed on the various plants mentioned.
In the field of Public Health the authors wrote that their work could lead to an environmentally friendly control of trypanosomatid threats to global health.

Response: We agree and have now qualified our conclusions to note the lack of field data, as described in response to the first comment of Reviewer 3 (above). We also composed two new paragraphs to describe (1) the relationship between the species tested and known sand fly sugar sources and (2) the chemistry of other known sugar sources. These new sections (i.e., the second and third paragraphs of the Discussion) are quoted in response to your comment re: Line 137 and known sugar sources.

Reviewer #2: Good

Reviewer #3: The compilation done here would be extremely helpful as a screening process to test experimentally these compounds in the insect vector. Such conclusion would be more restrictive, but certainly more accurate to the data presented.

Response: We have noted the need for this experimental work in response to your first comment above.

Editorial and Data Presentation Modifications

Reviewer #1: Lines 13 and 53: Insect-vectored Leishmania are the second-most debilitating of human parasites worldwide. The actual quote is "Among parasitic infections, this disease is responsible for the highest number of disability adjusted life years (a measure of health burden) after malaria." (Lymphatic Filariasis is surely more debilitating than cutaneous leishmaniasis caused by Leishmania major). While the authors repeat several times this health burden, it is somewhat over empathized.

Response: We rephrased the Abstract and the Introduction to better reflect the original source:

Abstract: "Insect-vectored *Leishmania* are responsible for loss of more disability-adjusted life years than any parasite besides malaria." **(Line 13)**

Introduction: *Leishmania…* "have a greater health burden (as measured by loss of disability-adjusted life years) than any human parasite besides malaria." **(Line 52)**

To avoid redundancy, we also deleted the phrase, "the second most disabling human parasite worldwide", from the first paragraph of the Discussion.

Line 54 The CDC numbers: For cutaneous leishmaniasis, estimates of the number of new cases per year have ranged from approximately 700,000 to 1.2 million or more. For visceral leishmaniasis, the estimated number of new cases per year may have decreased to <100,000. WHO numbers are less, so perhaps "reported" should be cautiously used.

Response: We qualified these numbers with the word "estimated" for both visceral and cutaneous disease:

"The sand fly-vectored *Leishmania* parasites are estimated to cause disease in >2 million humans each year, with 10% of the world's population at risk, and have a greater health burden (as measured by loss of disability-adjusted life years) than any human parasite besides malaria [3]. These infections include an estimated >0.2M infections cases of visceral leishmaniasis". **(beginning Line 51)**

Line 66: "floral nectar appears to be a preferred food source" Perhaps it should be noted that this study was carried out in a lush irrigated farming village surrounded by desert.

Response: We specified that the study by Muller and Schlein (2004) on attraction to nectar was conducted in an oasis: "Floral nectar appears to be a preferred food source, as evidenced by the attractiveness of flowering bushes in a desert oasis." **(Line 68)**

Line 137 and Discussion: Is there any evidence that sand flies feed actually on for example (Dicentra eximia, Brassica napus, Helianthus annuus, or Thymus vulgaris)? Examples of mesocosm would augment the meta-analysis. A real world example of this would be "DNA barcode for the identification of the sand fly Lutzomyia longipalpis plant feeding preferences in a tropical urban environment": Leonardo H G de M Lima et al Sci Rep, 2016 Jul 20;6: 29742 or if the authors comb the literature they can find Suaeda asphaltica as a sand fly food source or Cameron's work in South America on bean plants as actual sand fly food or Alexander B and Usma MC, study in Colombian coffee fields.

Response: Thank you for all of these references! They were helpful in expanding the Discussion to reflect on the relevance of our results to *Leishmania*-endemic regions. We now discuss the geographical limitations of our analysis, the related species on which sand flies have been shown to feed, and the potential flavonoid-mediated antiparasitic activity of non-floral sugar sources. The new text is quoted below:

"Our data suggest that around 20% of nectars contain flavonoids at strongly antiparasitic concentrations, although this number likely varies by region and season. Our analysis was focused on bee-pollinated species in the Northeast United States, where sand flies are absent, and therefore contained few of the specific plant species naturally used by sand flies in *Leishmania*-endemic regions. However, sand flies have been observed to prefer cultivated gardens (to which such plants could be introduced) over endemic vegetation [40], and have been associated with plants in the same families represented by the species analyzed here. For example, *Brassica napus* (Brassicaceae) nectar was among the highest in flavonoids; flowers of another member of this family (*Sinapis alba*) elicited feeding by *Phlebotomus papatasi* [48]. Nectar of *Impatiens capensis* (Balsaminaceae) had flavonoid concentrations (20.9 µM, all from strongly antileishmanial compounds) close to the median of the nectars examined (20.3 µM) and the median *Leishmania* IC50 (23.1 µM); branches of the congener *I. balsamina* were fed upon by *Lutzomyia youngi* in Colombian coffee plantations [49]. On the other hand, nectar flavonoid concentrations were considerably lower (1.6 µM total) in *Trifolium pratense,* the only Fabaceae species tested; plants of this family have been strongly associated with sand flies in field sampling [50] and DNA metabarcoding studies [51,52]. Flavonoid concentrations were also low (<1 µM) in *Cucurbita pepo* (Cucurbitaceae) and undetectable in *Catalpa speciosa* (Bignoniaceae), two other plant families associated with sand flies [40]. Based on these results, the amounts of antileishmanial flavonoids ingested by flies could vary substantially in different landscapes.

Besides floral nectar, other known sugar sources may also possess flavonoid-mediated antileishmanial activity. Flavonoid concentrations of sand fly-attracting fruits [16] appear similar to those found in nectar. Combined quercetin and luteolin contents ranged from unquantifiable in honeydew melon to 22.8 µM in nectarine, 33.1 and 39.7 µM in red and white guava, and 53.6, 84.1, and 91.1 µM in white, black, and red grapes respectively [53]. Honeydew from sapsucking insects such as aphids [12] likely also contains types and quantities of flavonoids similar to those found in floral nectar, based on the similar flavonoid profiles of honey from these two sources [54]. Further experiments are needed to assess the chemistry of local, fly-attracting, sugar-providing plant species and their effects on insect host and parasite mortality, as demonstrated for lectin-rich plant sugar sources in Israel [14,30,55]. Given that sand fly feeding on branches [56], flowers [12], and fruits [16] tends to be highly selective on a few local species, the scope of such research is likely achievable." **(beginning Line 179)**

Could it be that the compounds the authors are promoting would make better pharmaceutical products?

Response: Absolutely, plant extracts and compounds offer important leads for development of pharmaceuticals. For example, quercetin ameliorated infection in a murine model (Tasdemir *et al.* 2006, Antimicrobial agents and chemotherapy, doi: 10.1128/AAC.50.4.1352-1364.2006). We emphasize this extensive previous focus on pharmaceutical applications in the first paragraph of the Introduction:

"Due to their clinical significance, *Leishmania* spp. have been studied intensively in a search for affordable and effective treatments for human infections [5], including exhaustive testing of plant extracts and their components against both mammal- and insect-associated parasite life stages [2,6]. These studies have suggested new treatments for trypanosomatid-associated infections of humans [7] and related parasites of beneficial insects [8,9]." **(beginning Line 55)**

In this manuscript, however, we focused instead on the implications of these compounds for *Leishmania*-sand fly interactions and disease ecology, which we feel has received relatively little attention and deserves to be further explored.

While the MS is partly speculative in nature, the inclusion of some of the references add little to overall question.

Response: We added text to explain the inclusion of a discussion of incidental pollen ingestion by sugar-seeking flies:

"Feeding of sand flies on floral nectar may also result in incidental pollen exposure that, due to pollen's high flavonoid concentrations, has strong effects on *Leishmania* in the fly gut. Such incidental exposure was suggested by the high prevalence of Pinaceae DNA associated with sand flies at sites apparently lacking such plants [40]. This association was postulated to reflect exposure of flies to windblown pollen, which could also account for at least some of the DNA from *Cannabis sativa*—another wind-pollinated species not visibly present [40]. Introduction of pollen to nectaries by bees can increase nectar amino acid concentrations by an order of magnitude, and potentially introduce antiparasitic compounds from con- and heterospecific pollens as well." **(beginning Line 234)**

However, we shortened the remainder of this paragraph, removing two passages that were tangential due to their focus on pollinator health and ecosystem services:

"Given the apparent susceptibility of *Leishmania* to common nectar compounds at ecologically relevant concentrations, the high *Leishmania* infection intensity in flies from regions lacking in sugar sources [50], and the numerous nutritional benefits of floral resources and constituent phytochemicals for bees [53], landscapes containing plants rich in trypanosomatid-inhibiting nectars could mutually benefit pollinator and public health."

"Although currently a matter of speculation, the presence of pollinators could conceivably enhance the antiparasitic properties of nectar for disease-vectoring nectar consumers by introducing compounds from pollen or floral tissues [54,55]—a potential ecosystem service that is currently entirely unexplored."

Other references that may interest the authors for their discussion:

Lectins and toxins in the plant diet of Phlebotomus papatasi (Diptera: Psychodidae) can kill Leishmania major promastigotes in the sandfly and in culture. Jacobson RL, Schlein Y. Ann Trop Med Parasitol. 1999 Jun;93 (4):351-6.

Sand fly feeding on noxious plants: a potential method for the control of leishmaniasis Y. Schlein, R L Jacobson, G C Müller Am J Trop Med Hyg. 2001 Oct; 65(4):300-3

Response: Thank you for these references—they are indeed germane to this manuscript. Both are now included in the Discussion.

"Further experiments are needed to assess the chemistry of local, fly-attracting, sugar-providing plant species and their effects on insect host and parasite mortality, as demonstrated for lectin-rich plant sugar sources in Israel." **(beginning Line 203)**

Reviewer #2: Good

Reviewer #3: (No Response)

Summary and General Comments

Reviewer #1: The MS reviews the research of others on the in vitro screening of various extracts of nectar and pollen on Leishmania species and discuss the use of secondary metabolites in the control of the infections in the sand fly vector.
The inclusion of known plant food sources would improve the MS

Response: We agree and are eager to undertake such work. Given the timeline for revision, and because we are not currently permitted to conduct experiments in this study system, we have added qualifying statements throughout the text. These are detailed in response to the first comment of Reviewer 3 (R3: Methods).

We have also added a section to the Discussion to note cases where the tested plant species are in the same family as known sand fly food plants, and to note the flavonoid concentrations in several attractive fruits. This new section is quoted in response to the comment by Reviewer 1, re: Line 137 and sand fly food sources.

Reviewer #2: Phlebotomus take the nectar as nutrition. The effects of components in nectar are various. This study focuses on floral nectars and Leishmania transmission. The conclusion is that planting phytochemical-rich floral resources or phytochemically enriched baits could reduce Leishmania loads in vectors, providing an environmentally friendly complement to existing means of disease control. With these findings, there is a great hope to come over some diseases as Leishmaniasis without chemical solution, using natural environmental resources.

The manuscript how it is standing now, is well written, clear and shows good results to fight against the Leishmania. However, to improve the manuscript some points raised below need to be more detailed.

1- Line 117-118 (Ref 13), in this paper they have shown that some plant species reduce the transmission of malaria and other plants maintain the transmission, so, could you consider this in the discussion?

Response: We now end the Discussion by calling for more detailed modeling of the effects of sugar sources on *Leishmania* transmission, as demonstrated by Hien and colleagues for malaria:

"Empirical testing of these compounds in sand fly diets is necessary to confirm their efficacy in the insect vector and model the effects of sugar sources on parasite infection, vector longevity, and disease transmission, as was recently done for malaria [13]." **(beginning Line 258)**

2- In your references 2, 12, 17, 23 there is no DOI as well as in all your references in Supporting information 1, please add these details

Response: Thank you. We added these DOI's except for the original reference 12 (Müller and Schlein 2004, J. Vector Ecology), for which none could be found. We also updated the formatting of the references in the Supporting Information to include the DOI's for these as well.

3- Some species scientific names in your references are not italicized, you should correct that too

Response: We have now italicized binomial species names in the references.

Reviewer #3: The information discussed in this study is certainly interesting, but it is not being consistent with the broader potential speculated by authors. I suggest authors either support the study with some experimental tests of nectar compounds on insects or restrict claims to avoid the speculative character.

Response: We agree. Given the timeline for revision, and because we are not currently permitted to conduct experiments in this study system, we have added qualifying statements throughout the text. These are detailed in response to your first comment (R3: Methods).
