# Supplementary material for: Can floral nectars reduce transmission of Leishmania?
Source: PLoS Negl Trop Dis. 2022 May 12;16(5):e0010373. doi: 10.1371/journal.pntd.0010373 (PMC9098005; doi:10.1371/journal.pntd.0010373)
Supplement: Attachment — Submitted filename: leishmania_hypothesis_responses_v2.3.pdf [file pntd.0010373.s004.pdf]

Responses to reviewer comments

Many thanks to the three reviewers for their continued constructive criticism, which have helped us to continue to improve the manuscript. The original comments are listed in italics, followed by our responses and, when applicable, quotation of the text changed in the manuscript and its location. Line numbers refer to the unmarked version of the manuscript, rather than the one with changes tracked.

Because Reviewers #1 and #2 had no further suggestions, we address only the comments of Reviewer #3 below. Suggestions in this round of review were limited to the Conclusions, Editorial and Data Presentation, and Summary and General Comments.

Conclusions

Reviewer #3: In my view, the conclusion of this work still remain very broad considering the data based only in vitro tests to discuss a bigger question, a more restrictive statement would be more plausible.

Response: We understand the need to properly qualify our statements, given that the ideas presented in the manuscript remain untested either in parasite-infected insects or under field conditions. We have therefore expanded the Conclusions section to make clear the speculative nature of the current hypothesis and the conditions required for realization of the envisioned effects **(beginning Line 281)**:

> Our synthesis of existing data shows that sugar-seeking sand flies are attracted to floral resources, and that floral nectars contain antileishmanial phytochemicals at concentrations that inhibit replication of parasite cell cultures. The extent to which floral resources influence *Leishmania* epidemiology will depend on the contribution of nectar to sand fly diets and the extent to which *in vitro* inhibitory effects are realized in the guts of infected flies. If the effects of nectar on insect infection are commensurate with predictions based on nectar phytochemistry, reduction of transmission *via* supply of antiparasitic nectar sources in local landscapes—or phytochemical-based, transmission-blocking baits [16]– could positively influence public health.

Editorial and Data Presentation

Reviewer #3: I acknowledge all changes performed by the authors in the text, it improved considerably the manuscript, however I still would recommend some "major revision" in this work.

Response: Many thanks for your continued efforts to improve this work, which we have endeavored to apply to this second revision of the manuscript.

Summary and General Comments

Reviewer #3: Despite all modifications performed, the hypothesis raised in this work still consider a large knowledge in nectar compounds and their effect in the parasite in vitro, it has poor information related to the vector interaction, in which is essential considering to the potential claimed in this manuscript.

I still recommend authors discuss more about the physiology and behaviour of the insect that could justify the hypothesis. Even simple details regarding the insect could make all the difference and should be not selectively ignored. For instance, if the nectar amount taken by the insect would enough to affect the parasite considering the in vitro results presented here?

Please check here some papers that might help:

Ferreira, T.N., Pita-Pereira, D., Costa, S.G. et al. Transmission blocking sugar baits for the control of Leishmania development inside sand flies using environmentally friendly beta-glycosides and their aglycones. Parasites Vectors 11, 614 (2018). https://doi.org/10.1186/s13071-018-3122-z

Ferreira ME, de Arias AR, Yaluff G, de Bilbao NV, Nakayama H, Torres S, et al. Antileishmanial activity of furoquinolines and coumarins from Helietta apiculata. Phytomedicine. 2010;17:375–8.

Gontijo NF, Melo MN, Riani EB, Aleida-Silva S, Mares-Guia ML. Glycosidases in Leishmania and their importance for Leishmania in phlebotomine sand flies with special reference to purification and characterization of a sucrase. Exp Parasitol. 1996;83:117–24.

Jacobson RL, Schlein Y, Eisenberger CL. The biological function of sand fly and Leishmania glycosidases. Med Microbiol Immunol. 2001;190:51–5

Dillon RJ, EEl K. Carbohydrate digestion in sand flies: α-glucosidase activity in the midgut of Phlebotomus langeroni. Comp Biochem Physiol. 1997;116B:35–40.

Response: These are excellent resources; thank you for referring us to them. We acknowledge the importance of vector behavior and physiology in determining the extent to which sugar sources and their phytochemistry have the potential to influence parasite transmission. We have added details from the recommended articles to the Introduction, to better highlight the evolutionary and empirical evidence for the role of plant sugar meals and nectar specifically in sand fly biology. The expanded paragraph is copied below, with the revised text underlined **(beginning Line 62)**:

> The diets of blood-feeding, disease-vectoring insects such as sand flies and mosquitoes include ~~plant sugars~~sugar-containing plant tissues as well ~~as blood~~ [12]. Sugar sources differentially affect not only vector survival, but also the development of parasitic *Plasmodium falciparum* malaria in *Anopheles* mosquitoes [13] and *Leishmania major* in sand flies (*Phlebotomus papatasi*) [14], with effects mediated by secondary metabolites [15,16].~~. Different plant sugar sources can profoundly affect the development of *Plasmodium falciparum* malaria in *Anopheles* mosquitoes [13] and *Leishmania major* in sand flies (*Phlebotomus papatasi*) [14],[15].~~ Sand flies feed on plant sugars between acquisition and transmission of *Leishmania* to humans and other mammals [17], as ~~.~~demonstrated by caging flies with dye-infused branches, spectrophotometric detection of sugars or plant cell walls in the gut, and molecular analysis of field-collected flies showing the presence of plant DNA [18–20]. The importance of dietary sugars is evident from their effects on fly longevity. Flies survive less than a week under sugar source-poor desert conditions [21] and less than 2 weeks when reared on comparatively sugar-poor branches [22], but more than 7 weeks on 20% sucrose solution [22]. The abundance of sugar meal-inducible glucosidases expressed by the sand fly and by its *Leishmania* parasites provide additional

> evidence of mutual adaptation to an omnivorous lifestyle that exploits diverse plant sugars as food sources [23–25], and that sugar sources could be manipulated to control vectors and their parasites [16,22].
>
> The diets of blood-feeding, disease-vectoring insects such as sand flies and mosquitoes include plant sugars as well as blood [12]. Different plant sugar sources can profoundly affect the development of *Plasmodium falciparum* malaria in *Anopheles* mosquitoes [13] and *Leishmania major* in sand flies (*Phlebotomus papatasi*) [14],[15]. Sand flies feed on plant sugars between acquisition and transmission of *Leishmania* to humans and other mammals [16]. [17–19].dietary [20] [21][21][22–24]. Although sand flies may acquire their sugar meals from plant sap, fruit, or aphid- or cicada-derived honeydew [25], floral nectar appears to be a preferred food source, as evidenced by the attractiveness of flowering bushes and branches (relative to those soiled with honeydew) in a desert oasis [12]. The small (<1 µL) meal sizes of sand flies [22] would make the concentrated sugars found in nectar a profitable foraging resource, in spite of the small volumes available at each flower, explaining the general attractiveness of flowering plant food sources to sand flies and related dipterans [26]. The size of sugar meals is, however, impressive on a mass-specific basis—increasing the mass of females by >30% over 48 h [27]—consistent with the strong effects of meal chemistry on gut-dwelling *Leishmania*.

In addition, in the first revision, we added a Discussion paragraph to highlight the chemistry of food sources other than nectar **(beginning Line 214)**:

> Besides floral nectar, other known sugar sources may also possess flavonoid-mediated antileishmanial activity. Flavonoid concentrations of sand fly-attracting fruits [25] appear similar to those found in nectar. Combined quercetin and luteolin contents ranged from unquantifiable in honeydew melon to 22.8 µM in nectarine, 33.1 and 39.7 µM in red and white guava, and 53.6, 84.1, and 91.1 µM in white, black, and red grapes respectively [61]. Honeydew from sapsucking insects such as aphids [12] likely also contains types and quantities of flavonoids similar to those found in floral nectar, based on the similar flavonoid profiles of honey from these two sources [62]. Further experiments are needed to assess the chemistry of local, fly-attracting, sugar-providing plant species and their effects on insect host and parasite mortality, as demonstrated for lectin-rich plant sugar sources in Israel [14,15,21].

We also used the recommended references to highlight how sand fly digestion—specifically the action of glucosidases in the gut—could potentiate the activity of plant-derived glycosides (including flavonoids) against parasites by liberating strongly antileishmanial aglycones within the gut, as demonstrated by Ferreira *et al*. 2018. We now cite this article in the Approach section to justify our consideration of flavonoid glycosides **(line 123)**:

> Glucosidases have been shown to form antileishmanial aglycones from glycosylated coumarins in intestinal extracts [49].

In addition, we now emphasize the importance of fly digestive processes in formation of new compounds in the Discussion **(beginning Line 227)**.

> We focused on a limited subset of nectar components whose effects on *Leishmania* have been thoroughly studied, ignoring the effects of co-occurring chemicals that could also affect

> parasites (e.g., other flavonoids, lectins, and alkaloids), whether present in the ingested sugar source or formed during sand fly digestion (e.g., deglycosylation of coumarins or cyanogenic glycosides to compounds that reduce parasitic infection [63,64]).

The remainder of this paragraph introduces additional potential host-mediated effects **(beginning Line 231)**:

> Direct effects could be amplified by host-mediated reductions in levels of parasites due to phytochemical ingestion. For example, nectar-derived flavonoids stimulated immune gene expression in honey bees [65]; similar flavonoid-induced immune stimulation could enhance parasite clearance in flies. In addition, besides their effects on protozoa specifically, flavonoids are generally antimicrobial [37], and could inhibit growth of midgut bacteria that facilitate *Leishmania* infection [66]. It would be of interest to contrast the effects of similar flavonoid concentrations taken directly from plant tissues—which are delivered to the midgut—versus those from surface sugars (e.g., nectar and honeydew), which are first stored in the crop [57]. These differences in digestion could affect the phytochemical exposure of parasites found in different gut regions.

These additions should provide better context for the importance of specific sugar sources in the sand fly life cycle, the occurrence of flavonoids in sugar meals, and the role of fly digestion and other host-related processes in mediating the antileishmanial effects of ingested phytochemicals.
