# Supplementary material for: Can floral nectars reduce transmission of Leishmania?
Source: PLoS Negl Trop Dis. 2022 May 12;16(5):e0010373. doi: 10.1371/journal.pntd.0010373 (PMC9098005; doi:10.1371/journal.pntd.0010373)
Supplement: Attachment — Submitted filename: leishmania_hypothesis_respones_v3.1.pdf [file pntd.0010373.s005.pdf]

Responses to reviewer comments

Many thanks to the four reviewers for their continued constructive criticism throughout these three rounds of review, which have helped us to continue to improve the manuscript. The original comments from this round are listed in italics, followed by our responses and, when applicable, quotation of the text changed in the manuscript and its location. Line numbers refer to the unmarked version of the manuscript, rather than the one with changes tracked.

For brevity, we have omitted the complimentary comments to which no response was required.

Reviewer's Responses to Questions

Conclusions

Reviewer #3: Lines: 289-292. I would like just to point that some technical interspecies issues may need to be addressed here when extrapolating these results. It is unlikely that the phytochemistry from these floral compounds would be specific to sand flies. The ecological concerns would be similar to using synthetic compounds.

Response: We agree that these off-target effects deserve consideration. We added a sentence at the end of the Discussion to note their importance:

"In addition, the broader ecological consequences of floral compounds—whose effects are unlikely to be limited to sand flies alone—must be considered before implementation of interventions, particularly those that involve introduction of non-endemic plant species." **(beginning Line 280)**

Summary and General Comments

Reviewer #2: Good for me now, but they need to consider changing Phlebotomus langeroni in italic in reference 23…

Response: We have now italicized this species name in the reference list.

and the figure 1 in supporting information section is blurred

Response: We believe this is an artifact of the way the proof is compiled. These blurry images are in fact thumbnails of the main figures (not the supporting information), with links at the top of the corresponding proof page that allow download of the full .tif file.

To avoid this inconvenience, we pasted the figures at full resolution on line 511 of the manuscript, which we hope will facilitate easy review.

Reviewer #4: Revised version of the manuscript is significantly improved as helpful comments of reviewers were accepted. In my opinion, just few relatively minor changes are required:

Authors are aware that the sugar meal is directed first to crop (see lines 237-240). However, I am missing important information that only small droplets of sugar meal are released from the crop to the anterior (thoracic) midgut where the sugar meal is relatively quickly digested by glycosidases. This mechanism of sugar digestion may significantly decrease the effect of anti-leishmania compounds in the sugar meal. It is necessary to mention this fact either in Discussion or Conclusions.

Response: This is a key point that could limit the perfusion of the parasite's midgut niche with phytochemicals. We have replaced the last sentence of the paragraph on host-mediated effects of phytochemicals to emphasize this idea:

"The gradual release of nectar and honeydew from the crop to into the anterior midgut [57] could limit the exposure of midgut-dwelling parasites to phytochemicals from these sources." **(Line 239)**

(Previously, the sentence read: "These differences in digestion could affect the phytochemical exposure of parasites found in different gut regions.")

The above mentioned fact should be also reflected by more cautious deductions in the text, for example „would be" should be replaced by „might be" on line 267.

Response: We agree that this more cautious phrasing is appropriate, and have revised as suggested.

"This suggests that as little as 1% incidental addition of pollen to nectaries might be sufficient for *Leishmania* inhibition, even for nectars that lack antileishmanial flavonoids initially." **(beginning Line 266)**

Line 274: the proportion of microbiota in sand fly hindgut is negligible and unimportant in comparison to microbiota present in abdominal midgut of sand flies. Therefore, „hindgut microbiota" should be replaced by „microbiota in abdominal midgut".

Response: Thank you for pointing this out. We have revised as indicated:

"Parasites of this genus appear both sensitive to flavonoids and, given the parasite's establishment in the midgut and forward migration in the alimentary canal [17], directly exposed to ingested compounds before appreciable metabolism of these compounds by hosts or microbiota in the abdominal midgut." **(beginning Line 271)**
